# Cellular rejuvenation: molecular mechanisms and potential therapeutic interventions for diseases

**DOI:** 10.1038/s41392-023-01343-5

**Published:** 2023-03-14

**Authors:** Shuaifei Ji, Mingchen Xiong, Huating Chen, Yiqiong Liu, Laixian Zhou, Yiyue Hong, Mengyang Wang, Chunming Wang, Xiaobing Fu, Xiaoyan Sun

**Affiliations:** 1 Research Center for Tissue Repair and Regeneration Affiliated to Medical Innovation Research Department and 4th Medical Center, PLA General Hospital and PLA Medical College; PLA Key Laboratory of Tissue Repair and Regenerative Medicine and Beijing Key Research Laboratory of Skin Injury, Repair and Regeneration; Research Unit of Trauma Care, Tissue Repair and Regeneration, Chinese Academy of Medical Sciences, 2019RU051, Beijing, 100048 P. R. China; 2 State Key Laboratory of Quality Research in Chinese Medicine, Institute of Chinese Medical Sciences, University of Macau, Taipa, 999078 Macau SAR, China

**Keywords:** Senescence, Diseases

## Abstract

The ageing process is a systemic decline from cellular dysfunction to organ degeneration, with more predisposition to deteriorated disorders. Rejuvenation refers to giving aged cells or organisms more youthful characteristics through various techniques, such as cellular reprogramming and epigenetic regulation. The great leaps in cellular rejuvenation prove that ageing is not a one-way street, and many rejuvenative interventions have emerged to delay and even reverse the ageing process. Defining the mechanism by which roadblocks and signaling inputs influence complex ageing programs is essential for understanding and developing rejuvenative strategies. Here, we discuss the intrinsic and extrinsic factors that counteract cell rejuvenation, and the targeted cells and core mechanisms involved in this process. Then, we critically summarize the latest advances in state-of-art strategies of cellular rejuvenation. Various rejuvenation methods also provide insights for treating specific ageing-related diseases, including cellular reprogramming, the removal of senescence cells (SCs) and suppression of senescence-associated secretory phenotype (SASP), metabolic manipulation, stem cells-associated therapy, dietary restriction, immune rejuvenation and heterochronic transplantation, etc. The potential applications of rejuvenation therapy also extend to cancer treatment. Finally, we analyze in detail the therapeutic opportunities and challenges of rejuvenation technology. Deciphering rejuvenation interventions will provide further insights into anti-ageing and ageing-related disease treatment in clinical settings.

## Introduction

**Fig. 1:**
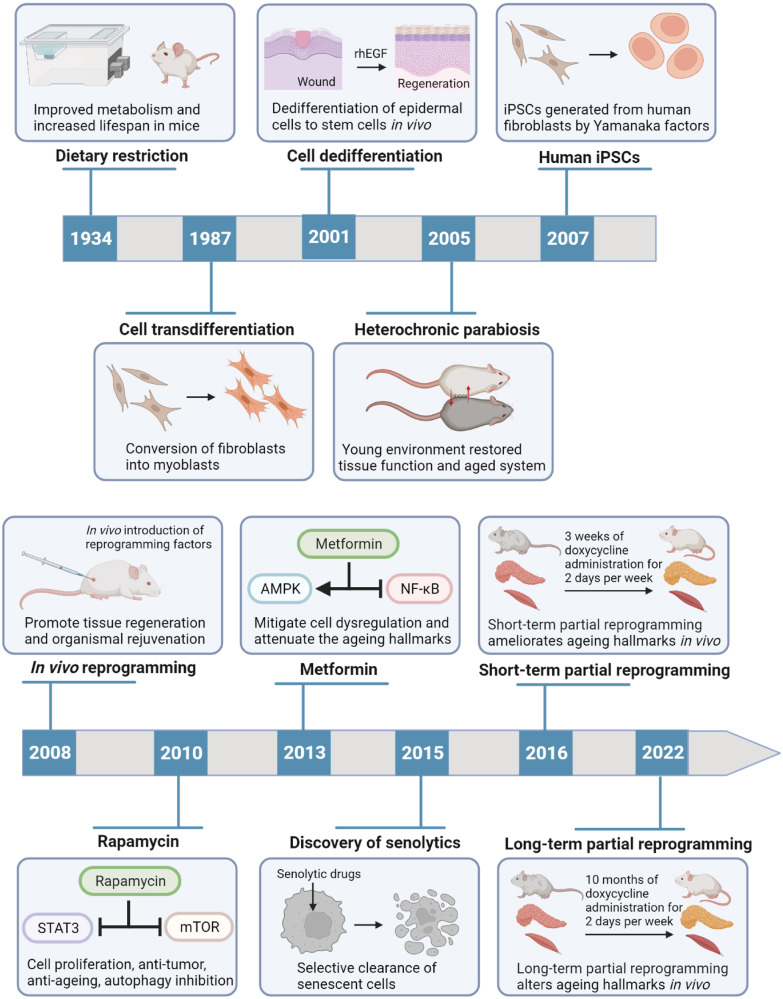
The milestone events for cellular rejuvenation research advances. Starting with the 1934 discovery of the influence of dietary restriction on lifespan extension, important findings on the subject of cellular rejuvenation are emphasized. More recently, the senolytics development and reprogramming technology have been widely applied for cellular rejuvenation. rhEGF recombinant human epidermal growth factor, iPSCs induced pluripotent stem cells, AMPK 5’-AMP-activated protein kinase, NF-κB nuclear factor-κB, STAT3 signal transducer and activator of transcription 3, mTOR mammalian target of rapamycin. Created with BioRender.com

Ageing is a dynamic and time-varying process, typically manifested by cell damage accumulation, degeneration of tissue and organ structure and function, and increased susceptibility to diseases.^1^ As the risk factor for human mortality, ageing is closely associated with many chronic diseases, like diabetes, Alzheimer’s disease (AD), chronic kidney diseases (CKD), cardiovascular diseases (CVD), and cancer.^2^ Therefore, enhancing the knowledge of ageing and the development of rejuvenation interventions are priority targets in biomedical research. Dietary restriction (DR) was found to prolong lifespan in mice and rats in 1934, and there are currently many emerging rejuvenation treatments to enhance health and lengthen lifespan, such as genetic, pharmacological, dietary, and lifestyle modifying approaches (Fig. 1).^3^ However, these breakthroughs were obtained in short-lived organisms from yeast model to mice model.^4^ Considering the complex anti-ageing mechanisms, it still takes a long translational phase to implement rejuvenation interventions into clinical applications.

Rejuvenation usually refers to giving aged cells or organisms more “youthful” characteristics through various techniques, such as cellular reprogramming and epigenetic regulation.^5^ Especially, the technique using induced pluripotent stem cells (iPSCs) in vitro via Yamanaka transcription factors is becoming increasingly proficient in the methodology and applications.^6^ Different organisms share certain molecular and cellular characteristics that are indicative of ageing, such as cellular senescence, epigenetic changes, telomere attrition, genomic instability, stem cell exhaustion, deregulated nutrient sensing, loss of proteostasis, mitochondrial dysfunction, and altered intercellular communication.^7^ Targeting these hallmarks of ageing is thus a growingly crucial field of research for the development of innovative cellular rejuvenation strategies. Nevertheless, these interventions have been lacking core criteria of rejuvenation in anti-ageing development and human applications.^8^ It is necessary to accurately define rejuvenation and characterize the therapeutic effects systemically. To understand whether the ageing can be rejuvenated, it also needs to uncover the common or distinct mechanisms underlying rejuvenation in different cell types that build each organ.^9^ In addition, a complete framework that describes various rejuvenation strategies should be established, contributing to cellular rejuvenation applications in human diseases. Herein, we provide a systematic and comprehensive discussion of cellular rejuvenation mechanisms and therapeutic interventions. An in-depth understanding of the pivotal roles will provide further insights into cellular rejuvenation in human disease treatment.

## Roadblocks and targets for cellular rejuvenation

### Intrinsic barriers limiting cell rejuvenation

#### Epigenetic alterations and genetic instability

**Fig. 2:**
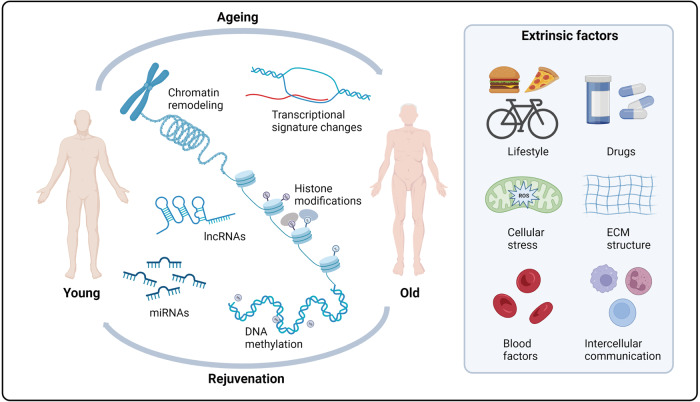
The epigenetic states of ageing and rejuvenation. Ageing and rejuvenation can be affected by intrinsic epigenetic alterations and genetic instability, like DNA methylation and chromatin remodeling. Moreover, many extrinsic factors, like microenvironmental cues, intercellular communication, and systemic factors, can also impact the epigenetic states of ageing and rejuvenation. miRNAs microRNAs, lncRNAs long noncoding RNAs, ECM extracellular matrix. Created with BioRender.com

Alterations in physiological and pathological ageing, are frequently caused by disruptions in genetic and epigenetic mechanisms. The universal definition of epigenetics is heritable genomic functionally modifications without DNA sequence variations. Gene expression and chromatin structure are connected with the major epigenetic alterations, which include DNA methylation, histone modifications, and noncoding RNA regulation. Defective transcriptional and chromatin networks have highlighted the contribution to cellular function, stress resistance, and ageing. Thus, epigenetic alterations and genetic instability might affect all cells and tissues in the anti-ageing process, but also provide opportunities for the design of novel rejuvenation treatments (Fig. 2).

##### Histone variants and modification

Histone variants serve as non-allelic counterparts of canonical histones and are also a common feature in aged organisms. H2A, H2B, H3, and H4 are four core histones variants with different propensity to diversity, regulating specific chromatin regions and gene transcription programs.^10^ Numerous research on ageing have demonstrated that histone variations in mammals are associated with a high abundance of macroH2A, H3.3, and H2A.Z.^11^ These results all suggested that the histone variants could be potential biomarkers for the ageing state.

Histone modifications include acetylation, methylation, phosphorylation, ubiquitylation, sumoylation, ADP ribosylation, deimination, and proline isomerization, depending on the modification process.^12^ These alterations occur at many sites and have various regulatory effects, including DNA repair, DNA replication, transcriptional control, alternative splicing, and chromosome condensation. Most prominently, histone methylation and histone acetylation play crucial roles in epigenetic alterations during ageing. Transcription is activated by the methylation of H3K4, H3K36, and H3K79, whereas it is repressed by the methylation of H3K9, H3K27, and H4K20.^13^ Furthermore, numerous investigations have shown that the control of organismal longevity and tissue ageing involves the manipulation of histone methyltransferases or demethylases.^14^ Therefore, various histone modifications might be important ageing-associated markers with the potential of anti-ageing drug screening targets.

##### DNA methylation clock

DNA methylation is widely appreciated as the reversible and inheritable epigenetic mark of the genome, participating in gene expression, biological regulation, and developmental processes in various eukaryotes.^15^ This common DNA modification is the synthesis of 5-methylcytosine (5mC), created when a methyl group is added to cytosine in a context involving a CpG dinucleotide. Long-term alterations in DNA methylation can be influenced by early-life environments and diet, increasing susceptibility to numerous diseases linked to ageing.^16^

Recent studies have discovered the mechanisms causing ageing-related DNA methylation changes and treated DNA methylation as the most promising biomarker of ageing.^17^ DNA methylation can directly facilitate transcriptome alterations in cells and tissues and also affect histone modification patterns to regulate gene expression during ageing. Borghesan et al. demonstrated that DNA methylation signatures were the biomarkers of healthy liver ageing and hepatocellular carcinoma progression, and the epigenetic synergism between DNA methylation and histone variant macroH2A1 could control the cancer cell escape from drug-induced senescence.^18^ Hence, controlling the DNA or histone methylation machinery might contribute to precise drug delivery. Moreover, certain CpG sites spread across the genome can be used to identify the mammalian DNA methylomes in order to determine the organismal biological age.^19^ DNA methylation-based ageing provides an appealing method of pathology prediction due to its continual readout of molecular changes in development. Many rejuvenation interventions, like calorie restriction, dwarfism, and rapamycin therapy, have been shown to slow down the epigenetic clocks and block some ageing-related changes in DNA methylation.^20^ Thus, the identification and confirmation of efficient anti-ageing therapies in humans have considerable potential in the DNA methylation.

##### Nucleosome remodeling

The nucleosome, consisted of pairs of core histones H2A, H2B, H3, and H4 and a DNA strand of 146 base pairs, is the basic unit of chromatin.^21^ In an ATP-dependent way, nucleosome remodeling controls DNA repair, replication, recombination, transcription, and cell cycle, as well as the expression of genes important for development and cellular functions in anti-ageing processes.^22^ The highly conserved ATP-dependent chromatin remodelers, referred to as sucrose nonfermenting 2 (SNF2) or switch/ SNF (SWI/SNF)-related enzymes, could utilize the energy released during the hydrolysis of ATP, in order to rebuild the chromatin.^23^ One particular ATPase might bind with other types of proteins to form distinct remodeling complexes. ATP-dependent nucleosome remodelers have important roles in modulating lifespan in organisms ranging from yeast to humans.

The modifications of histone core residues perform specific recruitment of transcription factors and remodeling complexes, and also shape nucleosome functions themselves.^24^ With increasing age, the nucleosome positioning and occupancy could be inevitably changed, including the loss of core histones and the substitution of canonical histones with variant histones.^25^ The availability of DNA for transcription factor binding was proved to depend on different combinations of histone modifications for chromatin remodeling, which also allows the histone code to modify gene expression with ageing.^26^ In addition, base excision repair (BER) is a vital DNA repair system for eradicating DNA lesions and maintaining the integrity of the genome.^27^ The deficient BER shows a strong link with ageing in human health. Nucleotide excision repair (NER) defects can also affect nucleosome remodeling, histone ubiquitination, stem cell reprogramming, and transcriptional activation, leading to ageing and developmental abnormalities in mammals.^28^ Therefore, DNA and histone contents within nucleosomes can go through chemical modifications that change the chromatin conformation and accessibility during ageing process.

##### Transcriptional signature changes

Numerous transcription factors that control growth, metabolism, and stress resistance are evolutionarily conserved. They are also involved in intricate interactions within various cell types for entire organismal physiologic development and ageing.^29^ During ageing process, there are many linked genes to prove the senescence phenotype or type.^30^ These biomarkers are linked with the cell-cycle arrest and SASP, such as increased expression of the cyclin-dependent kinase inhibitors p16 and p21, reduced expression of the nuclear lamina protein LaminB1, increased secretion of many inflammatory cytokines, and others. Many transcriptional responses to oxidative stress and pathogens decrease with ageing, owing to the declining function of the stress-responsive NF-E2-related factor 2 (Nrf2).^31^ Forkhead box O (FOXO) transcription factors are also revealed as critical mediators in crucial cellular processes in mammals during ageing.^32^ Thus, ageing was accompanied by both increased transcriptional instability and an accumulation of genetic errors. Besides, RNA N^6^-methyladenosine (m^6^A) modification has uncovered a new domain in post-transcriptional epigenetic regulation. In intervertebral discs (IVDs) degeneration, the m^6^A and the crosstalk between m^6^A and histone/DNA modifications have been proved to facilitate nucleus pulposus cellular senescence and aggravate cartilage endplate degeneration.^33^ To determine the diverse biological senescence, the transcriptome signatures associated with senescence and variability of the senescence program contribute to the identification of particular senescence biomarkers in tissues and organisms.

##### Noncoding RNA profiles

Noncoding RNAs (ncRNAs) are a broad and diverse group that produce non-protein-coding transcripts, including micro RNAs (miRNAs), long noncoding RNAs (lncRNAs), and circular RNAs (circRNAs). Several studies have discussed that the ncRNAs are able to bond to DNA, RNA, and protein, influencing cellular proliferation, quiescence, differentiation, apoptosis, and senescence.^34^ Specifically, ncRNAs can strongly implicate in controlling senescence at transcriptional, post-transcriptional, and post-translational stages. Especially, miRNAs are the best characterized small ncRNAs influencing ageing and lifespan. Multiple miRNAs, including miRNA-1, miRNA-145, miRNA-140, miRNA-34a, miRNA-106b, and miRNA-449a, are widely considered as critical regulators for cell senescence.^35^ They impact the SASP phenotype and modulate senescence through the classical p16, p53, and calcium signaling pathways.^35^ Furthermore, the lncRNAs expressions are known as a result of the disease-triggering stimulation in reduced cardiovascular vigor and cardiovascular ageing. There are increasing lncRNAs as indications of a deteriorating prognosis following cardiac events, such as the lncRNA MIAT, ANRIL, LIPCAR, and MALAT1.^36^ More importantly, the potential of circRNAs to serve as miRNA sponges and the interacting regulatory network between lncRNAs and miRNAs are all connected to ageing-related changes and the switching of the cell fate.^37^ In addition, extracellular RNAs in circulation and other bodily fluids also play vital roles within the context of ageing. The pericentromeric ncRNAs can be transported into neighboring cells via extracellular vesicles (EVs), and impaired the DNA binding of the CCCTC-binding factor to modify chromosomal accessibility and trigger an SASP-like inflammatory response.^38^

#### Macromolecular damage

The specific complex of cell macromolecules, including telomeres, proteins, and lipids, possesses intrinsically high resistance to modification, contributing to superior longevity in species. Reducing macromolecular damage is associated with an improvement in the majority of ageing-related physiological activities. Thus, the single macromolecular regulator of ageing and the interconnectivity among molecular phenotypes can both induce alterations in ageing-related phenotypes, limiting cell rejuvenation.

##### Telomere attrition

Telomeres are repetitive DNA sequences at chromosome ends. Telomerase adds repeats to the ends of the chromosomes during genome replication to counteract the loss of telomeric DNA. In most eukaryotes, the telomerase-based mechanism for telomere preservation is crucial for genomic stability and cell viability. The compaction of telomeric chromatin robustly protects the ends by limiting the accessibility of the DNA damage response machinery.^39^ However, telomeres are gradually shortened with cell division, and the cells enter a replicative senescence state when they cannot effectively guard the ends of the DNA.^40^ DNA damage accumulation with age also affects the genome randomly. Telomere dysfunction-induced foci and telomere-associated foci are two types of DNA damage that specifically target telomeres.^41^ Significantly short telomeres or changed telomere architecture can result in dysfunctional telomeres in ageing-associated pathology.^42^ This telomere dysfunction is closely associated with telomeropathies, telomere biology disorders, and telomere syndromes. In addition, ncRNAs also emerge as key inducers of telomere length maintenance in senescence and ageing-related diseases.^43^

##### Loss of proteostasis

Proteome homeostasis is maintained by the proteostasis network (PN), a macromolecular system that coordinates protein synthesis, folding, disaggregation, and degradation for organismal health and longevity. The autophagy-lysosomal system and the ubiquitin-proteasomal system (UPS) are two crucial mechanisms that regulate the turnover of organelles and aggregates.^44^ Proteasomes are also charged with removing normal and damaged proteins, participating in the evolutionarily conserved ageing mechanism and longevity regulation. However, ageing often shifts the balance between the protein lifecycle in organisms, resulting in pathology. UPS dysregulation occurs in the ageing process and several ageing-related diseases in mammals.^45^ PN component aggregation during ageing can elicit aberrant transcriptional procedures, reduced folding capacity, and the accumulation of misfolded species.^46^ This loss of proteostasis might further have profound consequences for ageing progression and age-related disease presentation. In addition, the increased ribosome pausing during ageing can also make the ribosome-associated quality control overloaded, leading to proteostasis impairment and systemic decline.^47^

##### Lipid Damage

Lipids are crucial components of all cell types, and perform various biological functions, including energy storage, cell membrane construction, signal transduction, protection, and mitochondrial regulation. There are a variety of bioactive lipids with critical roles in influencing cell age and the progression of several age-associated diseases and metabolic abnormalities.^48^ Specifically, the lipid assemblies act as scaffolding for the construction and function of signaling complexes and play critical roles in the preservation of proteostasis, thus involving in the ageing process and neurodegenerative disease development.^49^ Cholesterol, phospholipids, ganglioside GM3, and sphingomyelin are lipid classes that are typically present in cell membranes, playing their respective roles in membrane fluidity and rigidity. The lipid-induced changes in membrane structure and remodeling of lipid composition are the causative agents of ageing phenotypes.^50^ Moreover, many pathways regulating ageing and longevity are also linked to lipid metabolism and lipid signaling. According to research on the serum and plasma lipidome in centenarians, descendants of centenarians and elderly people without age-related disorders, the lipid signature profile altered with ageing.^51^ It also showed changes in antioxidant capacity, lipid peroxidation levels, and inflammation along with modifications in lipid metabolic pathways with ageing.

#### Metabolic imbalance

Age-dependent alterations in the transcriptomes, proteomes, and metabolomes of different organisms and tissues reveal the imbalance of metabolic homeostasis. Remodeling of metabolic signals and metabolites in ageing and the control of lifespan is caused by organelle malfunction, redox imbalance, and changed signaling pathways.^52^ Both environmental and generated endogenous toxicants by metabolism are major contributors to macromolecular damage and physiological dysregulation during ageing. The metabolic phenotyping of ageing mice revealed the involvement of the adiponectin, growth hormone, and cytokine pathways in autophagy, stress response, genome integrity, mitochondrial biogenesis, energy balance, inflammation, and infection control.^53^

The main mechanism to foster ageing is the malfunctioning of vital cellular organelles, including the autophagosomal-lysosomal network and mitochondria. With advancing age, there is a reduction in autophagy activity, autophagosome production rate, and lysosome fusion activity.^54^ Insufficient protective autophagy during ageing might cause damaged cellular components to accumulate and dysfunction of cellular organelles, leading to metabolic imbalance and further ageing. Moreover, defective mitochondrias produce insufficient ATP and frequently produce more ROS to enhance oxidative stress.^55^ Aged cells commonly develop mitochondria with aberrant characteristics, such as point mutations and mitochondrial DNA (mtDNA) deletions.^56^ In addition, mitochondrial metabolism also includes carbon metabolism (the tricarboxylic acid (TCA) cycle), the biosynthesis of Fe/S clusters, and the metabolic consequences of mitophagy.^57^ These metabolic processes are highly dynamic and all influence different facets of ageing.

There are metabolic enzymes and pathways to maintain homeostasis, including acetyl-coenzyme A (acetyl-CoA), pyruvate, 2-oxoglutarate, glycolysis, the TCA cycle, the urea cycle, respiration, and oxidative phosphorylation.^58^ The dysfunctions of these metabolic enzymes and mechanisms trigger metabolic disorders and restricted lifespan. Many core metabolites are appearing as key regulators of ageing, including nicotinamide adenine dinucleotide (NAD^+^), reduced nicotinamide dinucleotide phosphate (NADPH), α-ketoglutarate (α-KG), and β-hydroxybutyrate (βHB).^59^ A change in the NAD+/NADH ratio or the size of the NAD+ pool can cause the biological system to malfunction and result in a variety of metabolic diseases, ageing, and cancer.^60^ In addition, the telomere shortens and telomerase dysfunction might downregulate peroxisome proliferator-activated receptor gamma coactivator (PGCs) and other metabolically relevant genes, which are linked to hampered mitochondrial biogenesis and function, reduced gluconeogenesis, cardiomyopathy, and elevated ROS.^61^ It has been demonstrated that altering the insulin/IGF-1 and mammalian target of rapamycin (mTOR) signaling pathways significantly slows down the ageing process in a variety of species.^62^ Metabolic interventions, like time-restricted feeding, ketone bodies, rapamycin, metformin, resveratrol, NAD boosters, glycolytic inhibition, mitochondrial-derived peptides, and poly (ADP-ribose) polymerase (PARP) activators, all target these conserved pathways and biological ageing mechanisms across species, to boost adaptability, rehabilitation, and postponed ageing.^63^

### Extrinsic factors impacting cellular rejuvenation

#### Local microenvironmental cues

Extrinsic cues are transmitted by different stromal cell types within their niche and tissues, resulting in an actively responsive microenvironment. The ECM, neighboring cells, and signaling molecules, such as hormones, growth factors, and metabolic products, all play roles in mediating the interaction between the cell and the microenvironment. With time, the microenvironment that maintains multicellular organization is chronically altered, which further remodels the intracellular processes and induces ageing and cancer development.^64^

MSC lineage shift in ageing is mostly caused by microenvironmental effects. The crucial microenvironmental cues that cause differentiation abnormalities in MSCs are caused by hormonal, immunologic, and metabolic variables. For example, BMSCs in ageing could misdirect the differentiation toward adipocytes to impair osteogenesis, leading to the pathogenesis of osteoporosis.^65^ Especially, bone-fat reciprocity and the development of mesenchymal progenitors toward an adipogenic fate were mostly caused by the microenvironmental changes that occurred with in vivo ageing.^66^ Therefore, distinct microenvironmental conditions are deciding on cell fate, including exogenous growth factor stimulation, adjacent cells communication, pH, osmolarity, oxygen concentration, temperature, air pressure, biomechanical and electromagnetical influence.^67^ These microenvironment changes might increase the difficulty for normal cells to maintain homeostasis and react to damage.

#### Age-associated changes in ECM structure and composition

As the three-dimensional macromolecular network without cells, the ECM is primarily made of an interconnected system of fibrillar and non-fibrillar collagens, elastic fibers, and glycosaminoglycan-containing non-collagenous glycoproteins (hyaluronan and proteoglycans). Certain enzymes that stimulate ECM destruction, like matrix metalloproteinases (MMPs), mediate the ECM remodeling process.^68^ ECM maintains tissue integrity, and its dysregulation during ageing leads to various disease disorders by altering its composition, morphology, rigidity, and abundance. Many ECM genes and remodelers can be directly regulated by the mTOR signaling pathway, SIRTs, and numerous longevity-promoting transcription factors, such as KLF4, MYC, and HIF1, which control ECM dynamics during ageing.^69^

The ECM is necessary for normal tissue repair, but excessive deposition can cause organ malfunction and the onset of fibrotic and degenerative diseases. Especially, the adult dermis quality following complete maturation gradually deteriorates with age, such as atrophy of the elastic network, disintegration of collagen fibers, and alterations modifying proteoglycans.^70^ Moreover, ageing causes localized flaws and superficial fibrillation of the articular surface to accumulate. For instance, osteoarthritis (OA) might occur due to the altered matrix component composition, declining water content in the tissue, and increased catabolism in the ECM.^71^ Age-dependent functional deficits of muscle stem cells (MuSCs) are attributed to extensive ECM remodeling during ageing.^72^ In cancer, the main biochemical, physiological, and mechanical factors associated with ageing ECM promoted invasive and cancer-like activity in both healthy and malignant cells.^73^

#### Altered intercellular communication

Intercellular communication networks are essential for the coordination of biological processes in healthy and pathological settings of multicellular organisms. Senescence and ageing are influenced by interferences with intercellular communication caused by metabolic, mechanical, or biochemical triggers. To sustain physiologic function and respond to diseases, the many cell types that form the neurovascular unit (NVU) are in constant contact. The insufficient crosstalk between NVU cells impairs neurovascular coupling and blood-brain barrier dysfunction, thus leading to ageing and related neurological and neurovascular diseases.^74^ Moreover, there are also many organelle-organelle and organelle-cytosol communications impacting chronological ageing. These communications form an intricate network involving various movements of metabolites between cellular compartments. The process of stem cell ageing and tissue and organ functional declining is attributed to mitochondrial-ER crosstalk.^75^ Age-related diseases and the ageing process are linked to aberrant EV secretion and disturbance of the mitochondrial-lysosomal axis.^76^

Senescent cells are extremely proactive and interact with nearby cells through a variety of intercellular channels, including SASP. As the traditional soluble SASP, soluble factors, growth factors, and matrix remodeling enzymes are released. Intercellular communication during senescence via receptor or cell-ECM interaction is referred to as nonclassical SASP, and emerging SASP components include EVs.^77^ Furthermore, EVs are released into extracellular space and act as a cell-to-cell means of communication. EVs have negative impacts on downstream effectors at the levels of immunology, inflammation, gene expression, and metabolism in the ageing setting and age-related illnesses.^78^ Aged and senescent cells are proved to release more EVs than young cells.^79^ In addition, several environmental conditions, including air pollution, ultraviolet light, nutrition, and physical exercise, have been verified to impact the communication network via EVs, further impacting ageing.^80^

#### Systemic factors

Alterations in the systemic environment of cells and tissues play a role in the reversible process of ageing. Organ dysfunction with ageing is caused by blood-mediated cell-extrinsic alterations and important molecular mechanisms in the systemic environment. One of the cell types that reacts to young blood exposure is the hematopoietic stem cells (HSCs).^81^ The hematopoietic and immunological systems can be rejuvenated by the young transcriptional regulation system and cytokine-mediated cell-cell interactions in HSCs. Many agonists and antagonists of specific signaling pathways have the effective capability of resetting tissue stem cells in aged organs into rejuvenating state.^82^ In addition, systemic obesity, air pollution, exercise, and psychological stress have been clarified to accelerate ageing at molecular and epigenetic levels.^83^ Some biological techniques, such as heterochronic transplantation and parabiosis, might give cells and substances that are more abundant in young individuals to recover the function of aged tissue. Heterochronic parabiosis is the surgical method of young and aged organisms using a common vascular system, showing the significant impact of the systemic environment on ageing and rejuvenation. Many studies on neurogenesis have reported the pro-neurogenic “youthful” factors in the circulation and “ageing” substances that reduce stem cell activity in heterochronic parabiosis models of young and aged mice.^84^ Moreover, systemic transplantation of stem cells also displays the therapeutic potential of preventing age-associated degeneration. However, the systemic and hormonal changes with age, including pro-inflammatory cytokine profiles and sex steroid changes, also influence stem cell transplantation effectiveness.^85^

### Rejuvenating-targeted cells for organismal youthful state

#### Stem cells

The major types of stem cells include adult stem cells, embryonic stem cells (ESCs), and iPSCs created by activating Yamanaka factors from various somatic cells. They have the unique capacity for self-renew and multipotency, and can differentiate into tissue-specific terminal cell types. MSCs are pluripotent cells developed from adult stem cells. Many studies have demonstrated that MSCs produced from various sources, such as bone marrow, adipose tissue, and umbilical cord blood, and MSC-derived compounds slowed ageing process and improved age-related conditions.^86^ Tissue stem cells are found in particular local tissue microenvironments called “stem cell niches,” which support stem cell maintenance. Tissue stem cells play key roles in facilitating organic tissue renewal and performing regenerative responses to injury.^87^ ESCs can self-renew and differentiate into multiple cell types of ectoderm, endoderm, and mesoderm lineages.^88^ More importantly, the iPSCs modified from autologous sources also have ESC-like states and pluripotent potential. iPSCs transform patient-specific samples from early cells into developed target tissues, showing potential for age reversal within the organism.^89^ These stem cells are excellent rejuvenation targets and all have promising potential in regenerative medicine.

With increasing age, decreased stem cell functionality can lead to diminished organ function and prolonged tissue repair. Targeting the age-related molecular basis of stem cells might reduce the deleterious effects of ageing. There are many rejuvenating approaches based on aged stem cells, such as delayed fasting, gene expression modulation, medicinal intervention, and niche changes.^90^ However, Ho et al. found that some rejuvenating approaches had no observable renewed effects on aged HSCs and aged bone marrow niches.^91^ Some rejuvenation techniques might show temporary benefits, but show harmful long-term effects by prematurely diminishing the stem cell pool.^92^ Thus, it is also vital to strike a balance between the regenerative properties of stem cells and their potential to induce cancer.

#### Vascular and connective tissue cells

Endothelial cells (ECs) and smooth muscle cells (SMCs) are critical building blocks of blood channels and are negatively impacted by premature or typical ageing processes. In ageing process, dysfunctional ECs and endothelial progenitor cells (EPCs) occur abnormal metabolism, the development into mesenchymal phenotype, vascular detachment, and myofibroblast formation, resulting in fibrosis and organ dysfunction.^93^ ECs and other neurovascular cells are key cells in maintaining blood-brain barrier function. Dysfunction of pericytes, astrocytes, and endothelial cells increases blood-brain barrier permeability during ageing.^94^ The intricate biological process of targeted EC regeneration involves migration, survival, proliferation, tube formation, and restoring blood flow to the ischemic organs for tissue homeostasis. EPCs might be obtained from the bloodstream or niches within the vascular wall and restored by the ectopic production of mediators that prevent senescence and the onset of ageing-related traits.^95^ Thus, targeting the endothelium via regulating the senescence-induced gene expression and other emerging rejuvenation mechanisms is essential for homeostasis and tissue regeneration.

The major stromal cell type is the fibroblast, which regulates tissue morphology by depositing ECM, and promotes cellular and microenvironmental homeostasis by secreting soluble substances and signaling proteins. During the ageing process, fibroblasts lose contractility and exhibit an unbalanced production and degradation of ECM proteins, ultimately leading to reduced connective tissue stiffness and even age-related diseases.^96^ Activated fibroblasts and senescent fibroblasts secrete inflammatory cytokines with a different ratio, affecting complex reprogramming and wound healing in mice.^97^ Many studies have insisted that some anti-ageing compounds like triacetylresveratrol and cannabidiol, gene expression, signaling regulation, and cell-cell communication are all effective methods for targeting fibroblasts for rejuvenation, longevity, and health.^98^ Hence, as a cell type commonly used for iPSC reprogramming, fibroblasts are essential targets for rejuvenation techniques and regenerative medicine.

#### Senescent cells

Senescent cells (SCs) comprise a heterogeneous cell population because of their various cell-autonomous activation pathways and microenvironmental circumstances. Although cell-cycle arrested, SCs are still metabolically active and can perform various functions of the parent cells.^99^ At present, SCs, as organismal carriers of irreparable damage, are identified by the senescence-associated gene expression, SASP production, DNA damage, and β-galactosidase activity.^100^ MSC populations with a high number of SCs are found less productive during transplantation.^101^ The excessive accumulation and activity of SCs are also linked with chronic ageing and age-related illnesses, including atherosclerosis, cardiac and kidney dysfunctions, neurodegeneration, and pulmonary fibrosis.^102^ SCs are also able to trigger senescence in non-senescent cells. High quantities of SCs secreting chronically SASP are found in aged tissues, causing irreversible reprogramming of their adjacent cells.^103^ Hence, it also needs to prevent the subsequent development of SCs after the emergence of initial SCs. Partial reprogramming of SCs can reduce the persistent inflammatory state related to ageing and secondary senescence in surrounding cells by inducing the SASP.^104^ The specific gene expression on the surface of SCs might lead to the advancement of senolysis techniques for selective elimination.^105^

#### Immune cells

During ageing, the immune system progressively undergoes disorders of immune cell generation, differentiation, and function, leading to a chronically subclinical inflammatory condition. Several studies have proposed that targeting central immunological processes and specific immune subpopulations can reduce specific age-induced immune changes.^106^ Neutrophils are abundant immune cell populations in early injury and serve numerous functions in tissue regeneration. Macrophages reside in the bone marrow and are defective in efferocytosis and hyperactivated with ageing. Neutrophils can also function as an anti-inflammatory shift in macrophages by influencing the surrounding microenvironment or controlling the behavior of macrophages during tissue injury.^107^ The deficiency of immunosurveillance might hamper the SCs clearance and induce a microenvironment of chronic inflammation, leading to pro-tumorigenic events.^108^ Therefore, enhancing the immune surveillance ability of macrophages is also an effective rejuvenation target, due to the macrophage function of selectively SCs recognition and elimination.

The senescence in immune cells affects innate and adaptive immunity, particularly natural killer (NK) cells, B cell, and T cell function, potently driving age-related changes in solid organs. In vivo reprogramming might be significantly impeded by NK cells, which identify and eliminate partially converted cells in a degranulation-dependent mode.^109^ T cell generation is decreased because of thymic involution. Some hormones, signaling pathways, cytokines, and growth factors might display T cell reconstitution effects and reduce the negative effects of age-related T cell deficiency.^110^ Adoptive cell transfer of naive T cells can promote immunological responsiveness to new antigenic stimuli and limit the growth of pathogenic memory T cells.^111^ However, the quantity of naive T cell reconstitution required to boost immunological defense in ageing organisms still has quantitative constraints.^112^ Furthermore, B cell production also decreases with age due to the reduction of hematopoietic bone marrow. An in vitro B cell population with youthful characteristics and cellular reactivity to immunological stimulation can be revived after B cell depletion in elderly mice.^113^

#### Other somatic cells

Many specialized cells with different sources deserve further research for tissue and organ regeneration and rejuvenation. For example, in the vertebrate retinas, Müller cells serve as the primary supportive and protective glial cells. They can secrete various cytokines and exhibit the potential for self-renew and trans-differentiation into retinal neurons.^114^ Pathological ageing might impair β-cell function in the pancreas, thus causing the imbalance of glucose homeostasis in the organism. Targeting β-cell and restoration of function is of vital importance for effective therapeutic strategies.^115^ At present, there are emerging reprogramming strategies conversing differentiated somatic cells into another cell type. For instance, the astrocytes and pancreas exocrine cells can be respectively direct reprogrammed into neuroblasts and β-cells via lineage-specific transcription factors.^116^ Thus, these cells and related genes and pathways might facilitate the target-based gene delivery and development of effective rejuvenation approaches.

## Common or distinct mechanisms of rejuvenation

### Signaling pathways

**Fig. 3:**
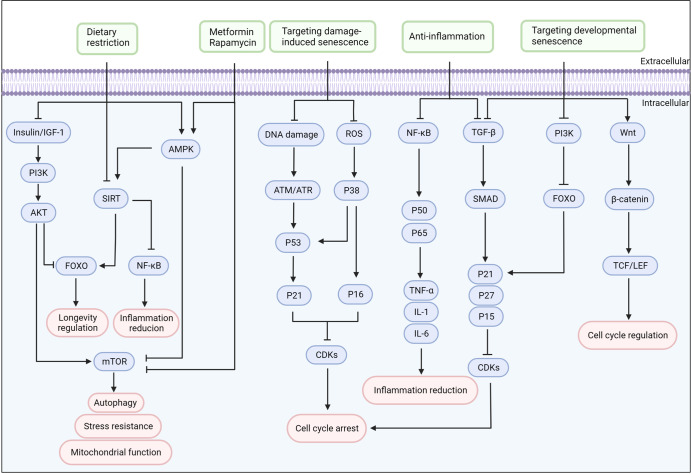
The target signaling pathways for cellular rejuvenation. Target signaling pathways for cellular rejuvenation are listed according to their biological functions. Many interventions, like dietary restriction and drugs, improve metabolism and extend longevity through nutrient-sensing pathways. In addition, targeting the pathways of damage-induced and developmental senescence can regulate the cell cycle and alleviate age-associated phenotypes. Modulating many inflammation pathways also provides an effective route to rejuvenation. IGF-1 insulin-like growth factor 1, PI3K phosphoinositide 3-kinase, AKT protein kinase B, AMPK 5’-AMP-activated protein kinase, NF-κB nuclear factor-κB, STAT3 signal transducer and activator of transcription 3, mTOR mammalian target of rapamycin, SIRTs sirtuins, FOXO forkhead homeobox type protein O, ROS reactive oxygen species, TGF-β transforming growth factor-β, CDKs cyclin-dependent kinase. Created with BioRender.com

There are various signaling pathways identified in the fields of ageing and rejuvenation, such as nutrient-sensing pathways, DNA damage pathways, ROS and mitochondrial unfolded protein response (UPR^mt^) pathways, inflammation-related pathways, transforming growth factor-β (TGF-β) pathways and Wnt/β-catenin pathways. The known role of these signaling pathways is complex and mutually connected. Given the prominent association of signaling pathways with rejuvenation and ageing, targeting these signaling systems pharmacologically and therapeutically has great potential for rejuvenation and human health (Fig. 3).

#### Nutrient-sensing pathways

Nutrient availability is crucial in regulating ageing and rejuvenation in mammals. Many growth factors, metabolites, amino acids, and carbohydrates can be recognized by several proteins, such as insulin/IGF-1, mTOR, SIRTs, and AMP-activated protein kinase (AMPK). The insulin/IGF-1 signaling pathway is an evolutionarily conserved glucose sensors mechanism, involving developmental defects and decreased adult functionality with age.^117^ The insulin/IGF-1 pathway participates in the regulation of many cellular functions, including the metabolism of lipids and carbohydrates, the cellular availability of glucose, gene expression, and cell differentiation, growth, and survival. The stimulation of insulin/IGF-1 receptors can stimulate STAT3 signaling via Janus kinase (JAK) and protein kinase B (AKT)-driven signaling pathways, then forming negative feedback to inhibit insulin/IGF-1 and induce cell immune senescence.^118^ The insulin/IGF-1 binding to membrane transporters can stimulate the phosphoinositide 3-kinase (PI3K)/AKT signaling pathway, followed by the downstream FOXO1 phosphorylation and mTOR upregulation, hence regulating cellular ageing and rejuvenation.^119^ Furthermore, the mTOR is also a conserved nutrient-sensing protein kinase that regulates eukaryotic cell growth and metabolism. There also remain AMPK/mTOR and PI3K/AKT/mTOR pathways implicated in the accumulation of unfolded and misfolded proteins and the regulation of the autophagy process during ageing.^120^ DR and rapamycin have been proved to inhibit the mTOR signaling pathway causing the downregulation of cell growth and lifespan extension.^121^ Hence, the insulin/IGF-1 and mTOR pathways are promising targets to suppress or delay ageing-associated diseases and extend lifespan.

SIRTs are a family of seven paralogous NAD^+^-dependent enzymes, that control cell proliferation, energy metabolism, stress tolerance, inflammation, circadian rhythms, neural function, and ageing.^122^ The activity of SIRTs in many or combinations of tissues might be necessary for lifetime extension and rejuvenation. Meanwhile, the AMPK pathway is a core mediator of energy homeostasis engaged in the pathobiology of ageing and age-linked disorders.^123^ AMPK is also considered as a crucial integrator of inflammation-controlling signals, including the inflammasome.^124^ It is well known that among different interventions to promote longevity, like exercise, intermittent fasting and DR, nutrient supply reduction activates AMPK to promote ATP production.^125^ Metformin can activate AMPK and SIRT1 and downregulates insulin/IGF-1 and mTOR, thus playing beneficial roles in energy metabolism and ageing.^126^ In general, the above nutrient-sensing pathways and the interaction between SIRT, AMPK, and mTOR all explain a mechanism for rejuvenation.

#### DNA damaging pathways

The cell functionality can be hampered by persistent DNA damage, which can also accelerate senescence and apoptosis.^127^ DNA damage response (DDR) pathway can protect against endogenous and exogenous damage and ensures the integrity of the genome.^128^ In response to stress, the established DDR contributes to the stimulation of p53 and p16 pathways and the depression of cyclin-dependent kinase (CDK) inhibitors to initiate and sustain the arrest of cell cycle.^129^ The genetic mutations that augment DDR and persistent DNA lesions can continuously activate the DDR. Moreover, the alterations in the integrity and efficacy of mtDNA repair contribute to DNA damage accumulation, illness, and ageing.^130^

The cytosolic DNA sensor cyclic GMP-AMP Synthase (cGAS) binds to the effector protein stimulator of interferon genes (STING), initiating a DNA sensing signaling pathway for innate immune responses.^131^ Misplaced cytosolic self-DNA and alteration of mitochondria structure and function can activate cGAS-STING signaling pathway, thus constituting age-related inflammation.^132^ The DNA mismatch repair system (MMR) is largely conserved across organisms and is essential for maintaining DNA integrity.^133^ Hence, MMR and related repair proteins are necessary for lifespan extension and rejuvenation targets. There are also a series of systems repairing the DNA, including BER, NER, and double-strand break (DSB).^134^ Thus, multiple interventions have been developed to lessen the DNA damage accumulation and alleviate age-associated phenotypes, such as lowering destructive molecules, restoring DNA damage, and responding to persistent DNA damage.

#### ROS and UPR^mt^ pathway

Reactive oxygen species (ROS) are mainly generated from oxidative phosphorylation in mitochondria, and maintain a dynamic balance with antioxidation systems under physiological conditions. Low ROS levels enhance the defensive mechanisms by producing adaptive responses for stress tolerance and longevity, whereas high ROS levels create insufficient adaptive responses that may accelerate the onset and course of ageing.^135^ The key mediators of the ageing process are ROS and ROS-induced oxidative damage produced by cellular metabolic and respiratory processes. Besides, mtDNA is susceptible to damage by mitochondrial ROS.^136^ Thus, the ROS causes oxidative stress, damages mitochondria, and induces energetic obstacles that lead to accelerated ageing and various diseases. Peroxiredoxins have been shown to facilitate ROS-based redox signaling and to trigger many cellular stress responses.^137^ However, these beneficial effects of ROS might be proved to be a sign of toxic adaptation.^138^ Interventions to ROS pathways are widely proposed as anti-ageing and rejuvenation strategies.

Metabolic stress, hypoxia, protein damage, and mitochondrial ROS all can impair mitochondrial protein homeostasis and functions. The transcriptional activation program of mitochondrial chaperone proteins and proteases is known as the mitochondrial unfolded protein response (UPR^mt^), which is a mitochondrial response to stress.^139^ Studies have shown that UPR^mt^ plays a significant role in many physiological processes and that its activation increases longevity and prevents ageing by regulating mitochondrial proteostasis.^140^ During mitochondrial dysfunction, there are many transcription factors necessary for the activation of UPR^mt^ genes in mammals. Activating transcription factor associated with stress-1 (ATFS-1) participates in the upregulation of genes involved in multiple stress response pathways for organismal survival of acute stressors.^141^ But, chronic ATFS-1 activation also has a negative effect on longevity. Meanwhile, in the initial development stages of elderly individuals, age-dependent levels of histone 3 methylation partially influence UPR^mt^ activation.^142^ UPR^mt^-mediated protective mechanism might be beneficial for rejuvenation mechanisms and therapeutics for diverse metabolic diseases and ageing-related disorders. However, prolonged UPR^mt^ activation might also induce the propagation of mitochondrial damage.^143^ The mitochondrial permeability transition pore in the inner mitochondrial membrane, can also initiate UPR^mt^ to promote ageing and age-related diseases.^144^

#### Inflammation-associated pathways

Multiple signaling cascades whose integration targets the induction of senescence, ageing, and associated disorders can be activated by the cycle of physiological interactions between inflammation and oxidative stress. In mammals, the major pro-inflammatory cytokines, which include interleukin-6 (IL-6), tumor necrosis factor-α (TNF-α), and IL-1α, significantly remodel the immune system.^145^ Damage-related stimuli cause the release of danger-associated molecular patterns (DAMPs) with ageing, and DAMPs that activate TLRs or the NLRP3 complex can be involved in sterile inflammation and age-related diseases.^146^ These age-related increases in chronic and low-level sterile inflammation are known as “inflammageing”, which shows a strong occurrence of various age-related diseases more than with ageing itself.^147^

The NF-κB signaling pathway is highly linked with the initiation and deterioration of tissue inflammation and ageing process. NF-κB can induce pro-inflammatory mediators, SASP, chemokines, and adhesion molecules, and the crosstalk between upstream signaling elements including MAPK, mTOR, and protein kinase B affects the transcriptional activity of NF-κB.^148^ Amplification loops for inflammatory processes are created when pro-inflammatory cytokines activate NF-B, which in turn can produce additional cytokines.^149^ The chronic activation of pro-inflammatory NF-κB/Rel and JAK/STAT signaling pathways contributes to the declined regeneration potential in mouse models.^150^ The JAK2 gene mutations in hematopoietic ageing can trigger abnormal involvement of downstream signaling pathways and the establishment of an inflammatory environment.^151^ Overactive JAK signaling is considered as a signature of immune disorders and has a significant impact on inflammation, coagulation, and thrombosis. Therefore, these shared pathways might provide a common route to rejuvenation by modulating inflammation, and some distinct pathways have great potential of targeting improving specific functions.

#### TGF-β signaling pathway

The transforming growth factor beta (TGF-β) superfamily is a vast protein group, including three TGF-βs (TGF-β1–3), bone morphogenetic proteins (BMPs), and growth differentiation factors (GDFs).^152^ The TGF-β family play roles through heteromeric combinations of type I and type II receptors, thereby activating many signal transducers, containing SMAD-dependent, SMAD-independent, and non-SMAD signaling pathways.^153^ These signaling pathways perform multiple functions in the development of embryo, tissue homeostasis and repair, immunological responses, tumor suppression, and metastasis. Many findings on age-related diseases have reported that TGF-β signaling dysfunction or increased levels of TGF-β ligands induce metabolic dysfunction, tissue fibrosis, inflammation, regeneration suppression, and cell degeneration.^154^ Besides, TGF-β signaling can induce specific epigenetic alterations further promoting senescence and ageing. Meanwhile, ageing also induces many abnormalities at the TGF-β receptor level. TGF-β signaling possesses dual functionality and versatility in some age-related disorders and cancer as a suppressor and a promoter.^155^ Hence, many strategies targeting TGF-β are mainly focused on inhibition of production, activation, binding to the receptor, and intracellular signaling.

TGF-β signaling can regulate matrix protein synthesis and matrix degradation, and alter cell-cell interaction. TGF-β overexpression results in ECM deposition, epithelial–mesenchymal transition (EMT), and cancer-associated fibroblast (CAF) formation, then leading to fibrosis and cancer.^156^ More importantly, dysregulation of the TGF-β/SMAD pathway is reported as an essential causative agent in tissue fibrosis, like hepatic, pulmonary, and cardiac fibrosis.^157^ TGF-β modulates several intracellular signaling cascades to deliver profibrotic effects, and the numerous ways of TGF-β interacting with other profibrotic pathways all offer the potential for therapeutic intervention.^158^ Furthermore, TGF-β signaling regulates the levels of angiogenesis-related molecules, like VEGF and CTGF, and mediates immune regulation, inflammation, and other pathways.^159^ In addition, GDF11, one cytokine of the TGF-β superfamily, is identified as a rejuvenating element in neurodegenerative and neurovascular diseases, such as the reversal of senescence and age-related variations, and the modulations of organ regeneration after injury.^160^

#### Wnt/β-catenin signaling pathway

Wnt proteins are secreted glycosylated proteins, which are cysteine-rich and can initiate the transcriptional co-activator, β-catenin, leading to the target gene upregulation via the family of T cell factor/lymphoid enhancer factor (TCF/LEF) transcription factors.^161^ By building a stable compound with the cell adhesion molecules of cadherin family, β-catenin, a transcription cofactor with dual roles, participates in cell adhesion.^162^ The Wnt/β-catenin signaling pathway participates in the modulation of genetic stabilization, cell proliferation, migration, and apoptosis for developmental processes and tissue homeostasis.^163^ The transcriptional results of Wnt/β-catenin pathway activation change with various cell types. The canonical Wnt/β-catenin pathway as the core mechanism is essential for directing stem cell regeneration and differentiation. For preservation and transition from the pluripotent state during embryo development, stem cells need β-catenin to moderate the response to Wnt signaling.^164^ Wnt/β-catenin signaling can also regulate the expression of telomerase reverse transcriptase (TERT) and change the telomere length, thereby affecting stem cells, ageing, and cancer.^165^ Wnt/β-catenin signaling can serve as a promising target to ameliorate the deterioration of stem cell function.

Research findings have verified that Wnt/β-catenin signaling regulates the ageing process of several tissues, performing different changes in different organs. Increased Wnt signaling has been found in aged organisms and excessive levels of Wnt are damaging to organism functionality.^166^ Wnt/β-catenin pathway is crucial for the growth and development of mineralized tissues, for the regulation of the skeleton in respond to loading and unloading, and for maintaining the viability and fitness of the adult and ageing skeleton.^167^ More importantly, the dysregulation of Wnt/β-catenin pathway is responsible for the fibrotic tissues associated with ageing, such as kidney, liver, lung, and heart fibrosis.^168^ Wnt/β-catenin signaling and TGF-β signaling can interact in the fibrosis process. Wnt/β-catenin superfamily members can be activated by TGF-β signaling and vice versa.^169^ Meanwhile, synaptic assembly, neurotransmission, and synaptic plasticity are all regulated by Wnt ligands, and neurodegenerative diseases are linked to deregulated Wnt signaling.^170^ Therefore, manipulating Wnt/β-catenin pathway might promote an efficient rejuvenation strategy versus ageing.

### Reprogramming-induced rejuvenation

#### Rejuvenation by induced pluripotent stem cells

iPSC reprogramming is widely defined as the rejuvenation of mature differentiated cells to an embryonic-like fate.^171^ By forcing the expression of specific transcription factors, induced pluripotency is the potent capacity to differentiate into all cell types. The transcriptome, epigenome, and metabolome of differentiated cells can be significantly altered by the transient expression of Yamanaka factors (OCT4, SOX2, KLF4, and cMYC; OSKM), which can also remodel the cells into iPSCs.^172^ The most common cell types for reprogramming to iPSCs are fibroblasts, which are greatly implicated in regenerative medicine and rejuvenation strategies. But in distinct subpopulations of fibroblasts, the change of fibroblast component and the level of secreted inflammatory cytokines might affect the in vitro reprogramming effectiveness and in vivo wound healing rate.^173^ In addition, the generation of iPSCs can realize the rejuvenation of MSCs and the iPSCs differentiation into desired cells via variations in DNA methylation, histone composition, and epigenetic models.^174^ Therefore, the iPSCs applications can minimize the genetic and epigenetic abnormalities associated with induced pluripotency. The iPSC reprogramming technique has extensive potential for molecular regeneration, disease modeling, and drug discovery.

The somatic cells of elderly donors can be utilized to generate human iPSCs, and cell reprogramming can reverse the key signs of ageing. By lengthening telomeres, reorganizing the mitochondrial network, alleviating oxidative stress, and recovering pluripotency, the reprogramming process transforms aged cells into young condition.^175^ iPSCs acquire ESC-like features, especially with similar mitochondrial properties, and can modulate mitochondrial or oxidative stress pathways leading to a state of rejuvenation.^176^ These transformation events can promote an extensive restructuring of mitochondria, including mitochondrial counts, morphology, viability, cellular metabolism, and the complexity of mtDNA. Telomere malfunction and chromosomal fragility can impair the ability of iPSCs to self-renew and the developmental pluripotency to differentiate.^177^ Telomere rejuvenation is an aspect of epigenetic reprogramming toward pluripotency and reprogramming can maintain or reverse telomere length and chromatin structure. Moreover, in the cells with telomere and mitochondria defects, somatic cell nuclear transfer (SCNT)-mediated reprogramming might be a better technology than current reprogramming factors.^178^ Therefore, the genetic foundation of ageing and rejuvenation can be modeled using iPSC lines, enabling the identification of new factors that prevent premature ageing or impact cell rejuvenation. In addition, iPSCs can be reprogrammed from patient cells via small molecules, miRNAs, and combinations of reprogramming factors, and be differentiated into somatic cells for drug testing and regenerative medicine.^179^

However, many studies clarified that ageing also might constitute critical barriers to cell reprogramming due to cellular senescence, inflammation, telomere reduction, and metabolic alterations.^6^ The aged or pathologic tissues are relatively inefficient at reprogramming and iPSCs derived from these tissue types might lack sufficient pluripotency and differentiation ability.^180^ Somatic cells produced from iPSCs might undergo premature senescence. The differentiated cells or premature termination of reprogramming can also carry the gene mutation and the full genetic heritage of the patient, which might contribute to long-term risk and tumor formation.^181,182^ Thus, the application of iPSCs needs efficient reprogramming and differentiation protocols, and the capability to maintain iPSC functionality in ageing microenvironment. Studies have proved that some longevity-promoting compounds and inhibition of age-related pathways enhance reprogramming in regenerative therapy. There are also many methods to boost the safety of iPSCs, such as using suicide genes to eradicate any undifferentiated iPSCs that remain after therapy, choosing younger donors, using appropriate cell sources, improving gene delivery techniques, replacing DNA delivery with proteins, mRNA, or regulatory miRNAs, using small-molecule DNA modifiers, and using low-passage iPSCs.^183^

#### Rejuvenation by lineage reprogramming

Lineage reprogramming, also described as direct reprogramming, is the procedure of switching somatic cells from one lineage to another with no transition for intermediate pluripotent states.^184^ This method of cell reprogramming generates particular cell types by ectopically expressing various lineage-specific transcription factors or miRNAs. For instance, recent research demonstrated that certain pro-neural transcription factors can directly reprogram non-neural somatic cells into neurons, skipping the pluripotent stage.^185^ Hence, lineage reprogramming techniques can be used to create a variety of cell types, including brain, cardiac, hepatic, and pancreatic cells. Meanwhile, because of the unique advantages of in situ conversion in live organs, lineage reprogramming is efficient and suitable for in vivo tissue repair and rejuvenation. Direct reprogramming in vivo may also benefit from minimizing hazards for genetic changes during prolonged in vitro culture, cancer development associated with de-differentiation, and immunological rejection following transplantation.^116^

The senescent program induced by ageing process and tissue damage can offer a beneficial microenvironment for in vivo lineage reprogramming. Chiche et al. suggested that tissue damage induced senescence and SASP secretion to promote the plasticity of resident cells, promoting in vivo reprogramming in tissue repair and regeneration.^186^ However, direct lineage reprogramming might retain epigenetic hallmarks of primary cells, like ageing hallmarks, which makes the reprogrammed cells suitable for modeling ageing-related disease.^187^ Thus, assigning a new cellular identity to terminally differentiated somatic cells, lineage reprogramming plays key roles in in vivo repair and rejuvenation. This technology can be built to utilize numerous and convenient autologous patient-derived cell types as a source, and is particularly crucial to replicate age-related traits and mimic the onset pathophysiology of diseases.

#### Rejuvenation by partial reprogramming

Cell reprogramming is a stepwise protocol. Studies have proved that somatic cell reprogramming mediated by OSKM for fewer than 7 days induced transient cellular alterations and reversible dysplasia, but partial reprogramming induction for more than 7 days could lead to tumor formation.^188^ Accordingly, the partially reprogrammed cells via short-term exposure to Yamanaka factors only partially lose their differentiated identity and undergo molecular rejuvenation without dedifferentiating to pluripotency.^189,190^ More specifically, partial cell reprogramming indicates that cells gain the ability to multiply and exhibit certain stem cell markers, but do not totally lose the cellular identity or receive all characteristics of pluripotent stem cells.^189^ Thus, partially reprogrammed cells have no risk of teratoma after transplantation. The cellular re-differentiation to the original phenotype with epigenome rejuvenation and the ability to react to optimum cocktails of certain differentiation factors is the representative feature of partial reprogramming.^191^

Generally, it is difficult to distinguish between the underlying epigenetic alterations that rejuvenate ageing cells and the changes that regulate the shift in cellular identity. Partial reprogramming is able to restore the common features of cellular ageing without altering the identity or function of the cells.^192^ Cyclic in vivo short-term induction of OSKM suppresses age-related phenotypes and histological alterations in different organs. Chondronasiou et al. demonstrated that partial and reversible reprogramming could improve the ageing states in cells, increase the ability of old mice to restore tissue damage, and lengthen the lifespan of progeroid mice.^193^ Potentially reversing the effects of ageing or tissue damage through organ-specific partial reprogramming could lead to the regeneration of the desired organ.^194^ In addition, partial reprogramming can generate a secretory phenotype that promotes cellular regeneration and improves the chronic inflammatory state linked to ageing and secondary senescence in nearby cells through enhancing SASP.^104^ Therefore, partial in vivo reprogramming can improve ageing-related traits, like the diminished capability to combat injury and the loss in the capacity of tissues and organs to regenerate during life.

### Epigenetic rejuvenation

#### Reset of ageing molecular signatures by epigenetic rejuvenation

Epigenetic remodeling is associated with biochemical modifications to the genome, leading to an altered response of gene transcription to physiological stimuli. DNA methylation clocks might detect a wide range of ageing-related epigenetic modifications that are indicative of genomic, cell biological, and tissue changes that occur during life.^195^ Epigenetic clocks can be more accurate than chronological clocks at estimating biological age, which aids in predicting human lifetime via age-reprogramming therapies. Protein-protein interactions can induce allosteric regulatory sites in complicated epigenetic machinery.^196^ Hence, accessing allosteric sites can assist in the development of epigenetic medicines with improved druggability and pharmacological characteristics. In addition, there are many lifespan-extending conditions, like Prop1^df/df^ dwarfism, calorie restriction, and rapamycin administration, slowing molecular variations linked to the epigenetic clock in mammals.^197^ Furthermore, reprogramming aged cells to a more youthful status carries the hazard of tumor formation. During reprogramming without de-differentiation, the mobility of heterochromatin protein 1β, an essential epigenetic modifier, has been proved to increase in SCs and promote epigenetic rejuvenation.^198^ The epigenetic rejuvenation with minimal de-differentiation can be realized by OSKM transduction in partial reprogramming. For epigenetic rejuvenation, distinguishing the rejuvenative features of reprogramming from dedifferentiation is a strong development.

#### Epigenetic regulation of mitochondria during rejuvenation

Mitochondria role in epigenetic processes mostly involves alterations in DNA methylation, histone modification in nuclear chromatin, and posttranslational gene control by noncoding miRNAs.^199^ The modulation of mtDNA and mitochondrial proteins by epigenetic and post-translational alterations contributes to the preservation of cellular health and homeostasis. Differential mtDNA methylation is associated with various conditions, including ageing and ageing-related diseases, changed metabolism, alterations in circadian rhythm, and even cancer.^200^ Thus, removing or counteracting the effects of mtDNA mutations in mitochondria might extend human health and lifespan. Moreover, the conserved histone lysine demethylases JMJD-1.2 and JMJD-3.1 could target the H3K27me2/me3 sites, which is also important for UPR^mt^ induction.^201^ MET2/ LIN65 histone methyltransferases were proved to mediate the chromatin remodeling and regulate the UPR^mt^-associated transcriptional networks.^202^ These findings revealed an epigenetic mechanism for regulating stress signaling and lifespan in response to mitochondrial abnormalities. Besides, all metabolic intermediates that serve as substrates or cofactors for epigenetic alterations originate from the Krebs cycle and other mitochondrial metabolic pathways.^203^ These metabolites contain acetyl-CoA, α-KG, S-adenosyl methionine (SAM), NAD^+^, and O-linked β-N-acetylglucosamine (O-GlcNAc) for DNA methylation and histone post-translational modifications, participating in controlling gene transcription and determining cell destiny.

#### Epigenetic regulation of retro-transposable elements during rejuvenation

Alternate splicing, different promoter or enhancer usage, ncRNAs, and epigenetic changes that impact the structure and function of chromatin all can modulate transcription. Retrotransposon-mediated promoters might also promote gene regulation and expand protein diversity for phenotypic variation and embryo development. During ageing, heterochromatin decay might upregulate the level of silent retrotransposons, leading to promoted mobility of retro-transposable elements (RTEs) within genomes and cellular homeostasis disruption.^204^ Chromatin of main retrotransposon classes, such as Alu, SVA, and long interspersed nuclear elements (LINEs), become relatively open in SCs and affect the evolutionarily recent elements, leading to increased transcription and ultimately transposition.^205^ Global hypomethylation of the genome can promote genomic instability and RTE activation, contributing to ageing.^206^ Hypomethylation of LINEs in cancer cells can restart the recruitment of many variant factors and is connected with an advanced disease stage and poor prognosis.^207^ DNA methylation can be oxidized by ten-eleven translocation (TET) enzymes as an aspect of the dynamic demethylation mechanism.^208^ TETs are responsible for LINE-1 demethylation in ESCs, but LINE-1s are negatively regulated by further TET-dependent activities. In nascent RNAs of human cells, m^6^A actively regulates the expression level of both autonomous LINEs and co-transcribed LINE relics, facilitating the retrotransposition of LINE.^209^

#### Epigenetic regulation of inflammation during rejuvenation

The inflammatory response can trigger epigenetic alterations, and epigenetics in turn can interfere with inflammation action. In reaction to severe inflammatory events, transitory activation of NF-κB-related innate immunity and senescence-related inflammatory elements might enhance reparative cellular reprogramming.^210^ The expression of numerous pro-inflammatory modulators is regulated by epigenetic procedures, which might therefore play a role in the progression of chronic inflammation. DNA methylation and histone acetylation are correlated with TNF-α expression during development and inflammatory disorders.^211^ Combinations of transcription factors maintain the identity of immune cells by controlling the hypo- and hypermethylation of cell-specific DNA. For instance, Sera et al. found that the X-chromosome-specific enzyme, UTX, maintained the expression of downregulated genes during ageing via demethylase-dependent and -independent epigenetic modulation, contributing to hematopoietic homeostasis and inflammation regulation.^212^ There are many phytochemicals and short-chain fatty acids regulating DNA methylation and histone modifications, participating in preventing chronic inflammation that worsens neurocognitive and cardiac performance and leads to metabolic disorders.^213^

#### Restoration of youthful functions in aged cells by epigenetic rejuvenation

Ageing is unavoidably accompanied by a diminished capacity to maintain tissue integrity and function. Cell or tissue rejuvenation without dedifferentiation is known as epigenetic rejuvenation, and it leads to a more youthful functional state and reversed ageing molecular markers.^214^ The central epigenetic regulatory mechanisms are based on the enzymes that modulate DNA and histones (methyltransferases, demethylases, acetyltransferases, deacetylases).^17^ The epigenome reprogramming can initiate ageing plasticity during heterochronic parabiosis, caloric restriction, or cellular reprogramming.^215^ These epigenetic modifications exhibit a strong capability of youthful function restoration in aged cells. In addition, epigenetic modifications can target several druggable pathways. In addition, senotherapy can increase lifespan, restore the functionality of bone marrow, muscle, and skin progenitor cells, enhance vasomotor function, and decrease the onset of atherosclerosis.^216^

### Metabolic manipulation

#### Mitochondria-based metabolic remodeling

Mitochondria is responsible for the ATP production required for organisms and apoptosis, autophagy, the creation of iron-sulfur clusters, amino acid synthesis, copper and lipid metabolism.^217^ The rates of fission and fusion govern the shape, size, and network of the mitochondria, which vary according to both internal and external cues like metabolism and stress.^218^ The metabolic condition can affect the form and function of mitochondria, consequently influencing organ function. Conversely, the disturbed mitochondrial dynamics, like genetic ablation of mitochondrial fusion and fission components, also cause metabolic changes. Age-related disruption in energy balance and an increased propensity for age-related illnesses may be caused by the reduction in mitochondrial activity. The crosstalk between mitochondria and other organelles like lysosomes might also lead to increased oxidative stress, reduced ATP production, and breakdown of cellular catabolic mechanisms, ultimately inducing metabolic imbalance and ageing.^219^

The mitochondrial functions in energy homeostasis and metabolism are closely associated with protein quality control factors in disease and age-related disorders, such as PTEN-induced putative kinase 1 (PINK1), Parkin, and TNFR-associated protein 1 (TRAP1).^220^ SIRTs control the mitochondrial metabolic checkpoint which can control stem cell maintenance and quiescence, and dysregulation of the checkpoint can deteriorate the function of aged stem cells.^221^ Aiming at the mitochondrial metabolic checkpoint might rejuvenate ageing stem cells and improve the functions of ageing tissue. Mitochondria also segregate many critical metabolic pathways, like the TCA cycle, fatty acid β-oxidation, and the one-carbon cycle. The synthesis of mitochondrial metabolites in these pathways might be involved in additional mechanisms that control stem cell activity and fate decisions.^222^ Moreover, the mitochondrial UPR^mt^ regulates many genes involved in protein folding, ROS defenses, metabolism, assembly of iron-sulfur clusters, and modulation of the innate immune response.^223^ Independent of the generation and aggregation of ROS, defective mitochondria also play a significant part in the ageing process. The Mitophagy pathway functions as a crucial mitochondrial switching that guides bioenergetic transition and metabolome remodeling attributes, to eventually define the effectiveness and quality of nuclear reprogramming and stemness transition in somatic cells.^224^ Mitophagy-induced rejuvenation of mitochondria governs the shift of bioenergetics and metabolome, hence facilitating a change in their capacity for cellular development. Thus, mitochondrial targeting or mitophagy regulation can promote metabolic remodeling, playing potential roles in rejuvenation and regeneration.

#### Oxidative stress

Deregulation of the redox state causes a rise of peroxides, ROS, and free radicals, which are collectively known as oxidative stress. Adaptive cellular responses to pathogenic challenges in ageing and age-associated disease tolerance, such as ischemia tolerance, can also be greatly benefited by moderate oxidative stress caused by diverse stressors.^225^ However, an imbalance between the generation of ROS and cellular antioxidant defenses can result in excessive oxidative stress, which accelerates ageing and the pathogenesis of illnesses like cancer.^226^

It has been demonstrated in numerous human cohorts and animal experiments that oxidative damage and inflammation might promote a state of susceptibility and raise the possibility of unfavorable health outcomes.^227^ Studies have suggested that the declined ability in response to oxidative stress with ageing is involved with the activated expression of Nrf2/EpRE signaling and its target antioxidant genes.^228^ Brahma-related gene 1 (BRG1) has been proved to protect cells from oxidative stress harm by encouraging the synthesis of antioxidants and inhibiting the generation of ROS.^229^ There are also many pathways, including MAPK pathway, PI3K/Akt pathway, heat shock proteins, p53, and NF-κB pathway, playing protective roles in combating oxidative stress for healthful ageing and longevity.^230^ Thus, these pathways might be effective mediators of oxidative stress for metabolic improvement and rejuvenation.

For oxidative damage regulation, bioactive exosomes have antioxidant effects on reducing the excessive ROS, promoting intracellular anti-oxidative stress defense, immunomodulation by blocking excessive ROS and changing mitochondrial function.^231^ Antioxidants targeting mitochondria, such as MitoQ and tiron, can penetrate the mitochondrial membrane and neutralize ROS at the core of the origin.^232^ Many compounds, like dibenzopyrone phenolic derivatives, caused the nuclear accumulation of Nrf2, stimulated Nrf2-governed cytoprotective gene expressions, and enhanced cellular antioxidant capacity.^233^ In addition, interventions including CR and exercise training targeted at restoring endogenous antioxidant ability and cellular stress reaction can contribute to successful vascular ageing and decreased risk for cardiovascular disease.^234^ In skeletal muscle, inflammation and oxidative stress are also the primary pathogenic features of ageing, and they are intimately linked to the onset and progression of sarcopenia. There are some promising antioxidant or anti-inflammatory substances, like minerals, vitamins, fatty acids, and antioxidant phytochemicals, to postpone skeletal muscle ageing and the onset of sarcopenia.^235^

### Autophagy modulation

Autophagy is a conserved, physiologic, and self-protective mechanism that supports cellular homeostasis and stress adaption. Autophagosomes with bilayered membrane vesicles can capture the degraded cellular components and subsequently merge with the lysosome to digest long-lived proteins, excess or damaged organelles, and misfolded or aggregation-prone proteins.^236^ There are three distinct forms of autophagy, namely macroautophagy, microautophagy, and chaperone-mediated autophagy. Depending on the selective autophagic degradation of several organelles, autophagy is subdivided into mitophagy, aggrephagy, pexophagy, reticulophagy, nucleophagy, lysophagy, xenophagy, lipophagy, ferritinophagy, and glycophagy.^237^ The autophagy process is important for maintaining cellular energetics, cellular reprogramming, organellar remodeling, immunity regulation, metabolism, and cellular survival.

Autophagy serves as one of the central pathways in the protection against functional loss and increased vulnerability to ageing process and age-related disorders. The activity of autophagy and the autophagy gene transcription by specific transcription factors, epigenetic changes, and microRNAs have emerged as crucially conserved pathways for promoting lifespan.^238^ In autophagy pathways, multiple points with age including autophagosome biogenesis, cargo loading, intracellular transport, and autophagosome-lysosome fusion or acidification have shown ageing or disease-associated deficits. Enhancing the function of the autophagy-lysosome system can eradicate age-related organelle degeneration, which might have regenerative benefits for cellular rejuvenation. The regulator of autophagy is the classical mTOR pathway and some mTOR-independent signaling cascades, such as MAPK-ERK1/2, STAT2, AKT/FOXO3, and CXCR4/GPCR, converge into PI3K signaling node.^239^ The histone deacetylase SIRT1 also regulates autophagy to influence ageing and age-related disorders.^240^ Furthermore, autophagy can eliminate the redundant production of ROS and maintain the cell proliferation capacity and regenerative ability.^241^ In addition, autophagy activation in ageing stem cells by genetic and pharmacological methods can improve cell properties and regenerative functions.^242^

### Activation of circadian clock

#### Basic molecular factors controlling circadian rhythms

The molecular circadian oscillator consists of transcriptional and translational feedback loops that are interlocked. BMAL1 and CLOCK (or NPAS2) have the ability to heterodimerize and bind to E-box elements of many clock-controlled genes to drive transcription.^243^ Early in the circadian night, PERIOD (PER) and CRYPTOCHROME (CRY) complexes are formed and suppress the transcription that is mediated by BMAL1 and CLOCK. As PER and CRY degrade over the night, negative regulation of BMAL1 and CLOCK is released, allowing the beginning of a new circadian day. These core clock proteins regulate 24 h oscillations in gene expression and activity, and at protein levels. In addition, the molecular clock controls the rhythmic expression of genes related to numerous cellular processes and nutrient-sensing pathways, which provides feedback to the primary clock system.^244^ The suprachiasmatic nucleus (SCN) in mammals serves as the body primary circadian clock, coordinating the timing of rhythmic activity with the light/dark cycle. Almost all the major genes implicated in the modulation of the circadian cycle have been found to induce alterations in circadian rhythms in their absence and to have an impact on health status.^245^ With increasing age, the transition toward morningness involves in epigenetics inside the molecular clock, modifications in the master clock, and downstream oscillator sensitivity to SCN signals.^246^ Many genes and proteins in circadian expression occur in phase shifts and decreased amplitude.^247^ The circadian body temperature, melatonin release, sleep-wake periods, patterns of locomotion, and drinking behavior can all vary with age. Thus, improving intracellular synchronization and the synchronization between SCN network and central and peripheral clocks might restore accuracy and stability to the ageing circadian system. Melatonin, produced in the pineal gland and mitochondria, participates in complicated intracellular signaling pathways with anti-ageing, antioxidant, chemopreventive, immunostimulatory, and tumor-inhibitory functions.^248^ SIRT1 modulation is associated with the activity of clock machinery and might resynchronize the dysfunctional cellular key clock circuits.^249^ Moreover, many environmental factors, like light exposure, lifestyle, and societal factors, can modify the circadian clock phase.

#### Circadian clock promotes stem cell rejuvenation

Stem cells contain a functional circadian clock whose rhythmicity contributes to the multipotent cell properties in constant renewal and injury response. Multipotent stem cells from various organs also have distinct clock gene expression profiles with various amplitude ranges.^250^ Meanwhile, dephased oscillators can provide stem cells the source of heterogeneity, to respond effectively to varied cues.^251^ Numerous embryonic and adult stem cell-dependent activities, including hematopoietic progenitor cell migration, follicular cycle, osteogenesis, regenerative myogenesis, and neurogenesis, have been linked to rhythmic oscillations and circadian clock regulation.^252^ The regulation of stem cell division and differentiation by the circadian clock is crucial for adult tissue regeneration. Histone alterations, DNA modifications, non-coding RNAs, huge multisubunit chromatin remodeling complexes, and additional epigenetic changes are also significant points in the circadian modulation of stem cell destiny.^250^

With ageing, stem cells receive signals from endogenous and external factors operated through circadian rhythms and epigenetic clocks. The circadian output and oscillator system have adapted to the particular homeostatic requirements of the adult stem cell region in the young organism. But the circadian functions of ageing stem cells might switch toward a stress-dominated program.^253^ Liang et al. found that the overexpression of CLOCK could rejuvenate physiologically and pathologically aged human MSCs.^254^ Mechanically, nuclear lamina proteins and KAP1 formed complexes with CLOCK, which preserved heterochromatin architecture and stabilized repeated genomic regions. Hence, circadian clock regulation provides opportunities to rejuvenate stem cells for various tissue engineering approaches.

#### Tissue-specific circadian clocks influence organ rejuvenation

The oscillations of peripheral clocks in numerous peripheral tissues, including the heart, liver, adipose tissue, retina, and multiple brain regions, can be controlled by synchronizing the circadian clock produced by SCN neurons.^255^ Meanwhile, there is a portion of transcripts expressed cyclically in each peripheral tissue, controlling the function of peripheral tissues.^256^ These tissue-specific circadian genes govern biological processes necessary for the preservation and dynamic modification of organ functions during the circadian cycle. Cell-autonomous circadian oscillations also have a substantial impact on the physiology and pathology of peripheral organs.^257^ In addition, the diurnal pattern of the light-dark cycle has a significant effect on the central SCN clock, while the feeding-fasting rhythm constrains the circadian rhythm of peripheral tissues.^258^

Many studies have suggested that circadian rhythms might be in a noticeably different phase during development in one cell or tissue type compared to another part of the body.^259^ Circadian programs are tissue-specific and even varied in the same tissue under distinct physiological states.^260^ During ageing, diminished or asynchronous circadian oscillations can alter signaling networks and tissue-specific gene expression profiles. These alterations in tissue-specific clocks might affect immune hyperactivation with ageing.^261^ Thus, the tissue-specific targeting of clock subunits by pharmaceutical techniques might aid to the treatment of age-related diseases and recover circadian coherence with a chronically disrupted clock. Besides, tissue susceptibility and reactions to toxicity also change during the circadian cycle, which indicates the development of drug timing administration.^262^

## Designing strategies to promote youthful states in cells and organisms

**Fig. 4:**
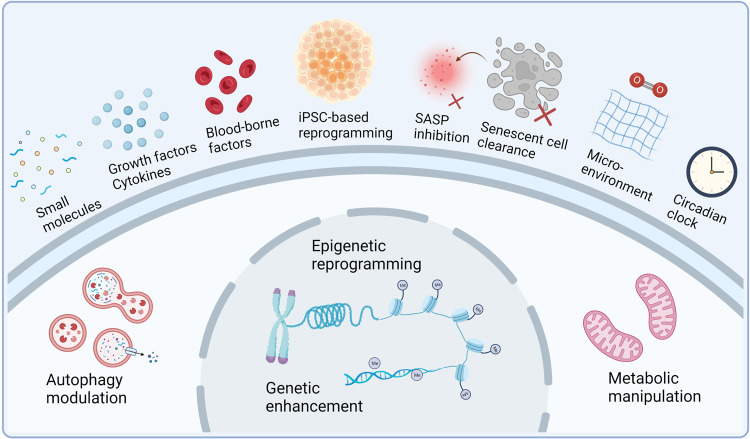
Schematic overview of strategies for cell rejuvenation. Various strategies have been developed for cell rejuvenation that leverage intrinsic and extrinsic factors, including epigenetic reprogramming, genetic enhancement, autophagy modulation, and metabolic manipulation. Furthermore, small molecules, growth factors and cytokines, blood factors, iPSC technology, clearance of senescent cells and SASP, microenvironment regulation, and circadian clock modulation can also exert great influences on cell rejuvenation. iPSCs induced pluripotent stem cells, SASP senescence-associated secretory phenotype. Created with BioRender.com

**Fig. 5:**
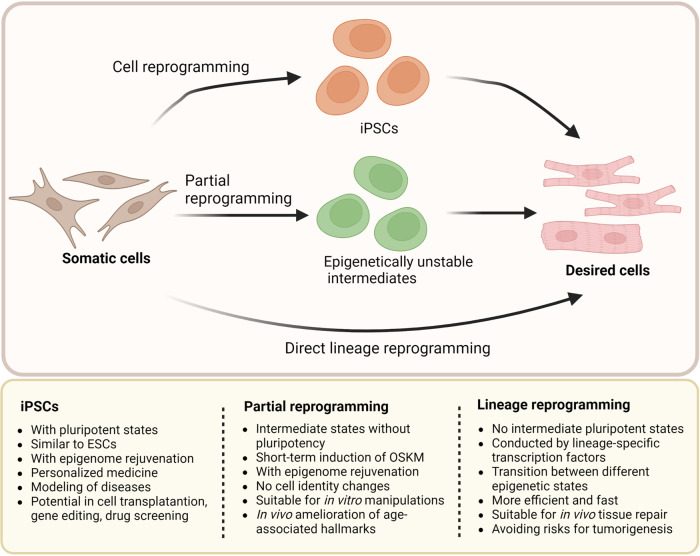
Reprogramming approaches for rejuvenation. The iPSC-mediated cell reprogramming is a protocol that somatic cells are first dedifferentiated into iPSC and then differentiated into the desired somatic cells. Partial reprogramming refers to a short exposure to Yamanaka factors only generates intermediates with high plasticity. Transforming somatic cells from one lineage to another without transitioning through intermediary pluripotent stages is known as direct lineage reprogramming. iPSCs induced pluripotent stem cells, ESCs embryonic stem cells; Oct3/4, Sox2, Klf4, c-Myc, OSKM. Created with BioRender.com

The molecular mechanisms that mediate cellular and organ ageing provide therapeutic targets for cellular rejuvenation. The increasingly rejuvenative approaches have been developed, and the schematic overview of strategies for cell rejuvenation is shown in Fig. 4. It contains cellular reprogramming, clearance of SCs and SASP inhibitor, metabolic manipulation, the restoration of aged stem cell function, microenvironment remodeling, resetting the circadian clock, immune rejuvenation, and heterochronic parabiosis. Especially, cellular reprogramming can rejuvenate the terminally differentiated cells to the pluripotent state or epigenetically unstable intermediates, and also to another desired cell type for tissue repair. The reprogramming technology mainly includes iPSCs, partial reprogramming, and direct reprogramming (Fig. 5).

### Resetting epigenetic clock in aged somatic cells by reprogramming strategy

#### Reset epigenetic clocks by reprogramming to a fully pluripotent state

The biological age of our cells, tissues, and organs is determined by an epigenetic clock based on alterations in the DNA methylation profile. A change in permission for future development and a return to a more flexible and pluripotent state are made possible by the removed DNA methylation instruction, and the production of iPSCs can also get around epigenetic constraints and change DNA methylation patterns to support youthful states in cells.^263^ According to the extensive DNA methylation analysis, the aberrant hyper-methylation spread randomly over the genome during the long-term iPSC reprogramming and then gradually diminished. These cells eventually adapted to closely resemble ESCs.^264^ Another study that used global DNA methylation and hydroxymethylation analyses demonstrated that DNA demethylation was a miR-29a depletion-mediated process during early reprogramming, and iPSCs produced from miR-29a depletion were epigenetically more similar to ESCs.^265^ The reprogramming technology to generate iPSCs has matured greatly. iPSCs can be derived from different tissue-specific cell types, such as human endometrial cells, endothelial cells in the placental artery, amnion-derived cells, fibroblast, blood cells, and even cancer cells.^264,266^ In the terms of differentiation degree, terminally differentiated cells and adult stem cells also could be reprogrammed into iPSCs. Senescent and centenarian cells are revived through pluripotent reprogramming, and the iPSCs produced from these cells have reset telomere length, gene expression profiles, oxidative stress, and mitochondrial metabolism.^267^ In addition, the fact that these SCs-derived iPSCs can re-differentiate into fully rejuvenated cells further demonstrated that reprogramming is not constrained by cellular senescence and that age-related cellular physiology is reversible.^267^ There are a variety of strategies to produce iPSCs, including viruses-mediated gene manipulation (e.g., adenoviral gene delivery), non-integrating episomal plasmids, proteins cell membrane-penetrating, the direct transfection of RNA and chemical reprogramming induced by small molecule compounds.^268^ The evolution of iPSC technology is around the improvement of reprogramming efficiencies and the avoidance of gene risk. Currently, given that each random integration event of a retrovirus poses a possible genetic risk, despite some of these approaches have produced lower efficiency than conventional reprogramming of retroviral administration, they constitute a step closer to therapeutic application.

#### Reduction in epigenetic age by partial reprogramming

Using partial reprogramming, the epigenetic age of cells can be reduced without losing cell identity, suggesting that full reprogramming of iPSCs is not necessary to reverse ageing of somatic cells. The previous study reported that OSKM + LIN28-mediated partial reprogramming in senescent human fibroblasts could result in the recovery of the high mobility of histone protein 1β, a feature characteristic for young fibroblasts.^198^ Likewise, multiple cellular features of dermal fibroblasts from middle age donors were rejuvenated following partial reprogramming with forced expression of OSKM, including transcriptome, epigenomes such as histone methylation and DNA methylation, and functional rejuvenation.^269^

Using a non-integrative partial reprogramming protocol with a cocktail of mRNAs carrying OSKM + LIN28, naturally aged human fibroblasts and endothelial cells exhibited a multifaceted alleviation of cellular ageing, such as resetting the epigenetic clock, reducing inflammatory responses in chondrocytes, and restoring youthful regenerative responses to age, in each case without changing cellular identity.^270^ Besides, OSKM-mediated partial reprogramming also restored youthful gene expression in adipocytes, MSCs, and myogenic cells, and the identity trajectory mapped by single-cell genomics revealed the temporary suppression of somatic identity programs.^271^ Partial reprogramming emerges as a powerful tool for the restoration of the cell youthful state.

#### In vivo reprogramming in ameliorating age-related physiological alternations

In vitro cellular reprogramming has observed alleviation of age-associated phenotypes and the reset of age clock. Due to the physiological complexity of the ageing process, it is necessary to study in vivo reprogramming to gain a deeper understanding of how it can affect cellular and organismal ageing. Ocampo et al. reported that transient cyclic induction of OSKM in vivo ameliorated age-associated hallmarks, extended lifespan in progeroid mice, and promoted tissue repair from streptozotocin-induced pancreatic damage as well as cardiotoxin-caused muscle damage, with no resulting teratoma formation.^272^ In the physiological ageing mice, the rejuvenating effects of long-term partial reprogramming are manifested in different tissues, including the skin and kidneys, as well as at the organismal level, and such effects were related to a reversion of the epigenetic clock and metabolic and transcriptomic changes, containing the decreased expression of genes mediating inflammation, senescence, and stress response pathways.^273^ Similar studies in naturally aged mice also reported that a single period of OSKM expression can alter epigenetics, transcriptomes, and metabolomes, resulting in a younger configuration in various tissues and in the serum, such as the reversal of DNA methylation changes and transcriptional changes, and the restoration of some ageing-related serum metabolites and biomarkers to young levels.^193^ In vivo reprogramming may represent an avenue for facilitating tissue repair. For example, expression of OSKM specifically in hepatocytes can dedifferentiate adult hepatocytes into progenitor cells and promote cell proliferation concurrently, and functionally, short-term in vivo reprogramming increases liver plasticity and promotes regeneration.^274^ Heart-specific in vivo reprogramming also induces adult cardiomyocytes to a fetal state, conferring regenerative capacity to adult hearts. and the short-term OSKM expression reduces myocardial infarction-induced damage and improves cardiac function.^274^ In vivo reprogramming might also hold a great promise against age-related diseases. In vivo reprogramming with short-term cyclic expression of OSKM also inhibits the progression of intervertebral disc degeneration (IDD), and significantly improves senescence-associated phenotypes in ageing nucleus pulposus cells (NPCs).^275^ Exception for OSKM, in vivo cyclic expression of the Forkhead box protein M1 (FOXM1) also ameliorates natural and progeroid ageing phenotypes and increases health span.^276^ Despite the great progress of in vivo reprogramming for cellular rejuvenation, there are some problems and limitations facing us. The first is teratomas, a type of tumor that could be cancerous, which may be attributable to too much OSKM induced by inappropriate dosage.^277^ Then, in vivo reprogramming is not enough for systemic rejuvenation, because the reprogramming factors fails to be delivered to certain tissues, which also explains why some study switched their focus to local rejuvenation such as NPCs.^275^ Finally, several ageing hallmarks such as nuclear and mitochondrial DNA mutations, metabolic aggregates in the cell and extracellular matrix, cannot be reset through in vivo partial reprogramming.

#### Epigenetic drugs

Currently, “epigenetic drugs” such as those that modulate enzymes that cause epigenetic changes are being considered as potential treatments against ageing. DNA methylation-targeted drugs mainly contain DNA methyltransferase inhibitors, like 5-Azacytidine. The removal of DNA methylation is a prerequisite for epigenetic rejuvenation and the generation of iPSCs.^265^ 5-Azacytidine was used to facilitate the reprogramming of human fibroblast into iPSCs by overcoming epigenetic barriers.^268^ Besides, brief methylation inhibition with 5-Azacytidine could confer human dermal fibroblasts a relaxed chromatin structure and short plasticity, and the 5-Azacytidine-treated fibroblasts exhibited the upregulation of pluripotency gene expression and differentiation capacity towards other germ layers.^278^ Bromodomain and extra-terminal inhibitors (BETi) that work to repress transcriptional elongation of acetylated chromatin are also for stem cell rejuvenation. BETi has exhibited the effect to induce keratinocytes dedifferentiation, to enhance the migratory ability in keratinocytes in vitro, and to promote skin wound healing in vivo.^279^ Then, the high throughput chemical screening discovered I-BET151 can robustly contribute to the expansion of PDX1^+^NKX6.1^+^ pancreatic progenitors (PPs), and these expandable PPs (ePPs) maintain pancreatic progenitor cell status in the long term and can efficiently differentiate into functional pancreatic β (ePP-β) cells, which optimize the stem cell therapy in the amelioration of diabetes.^280^ Histone methylation inhibitors have been reported to re-activate stemness-related genes. The 3-deazaneplanocin A (DZNep) compound, which specifically inhibits H3K27me3 and H4K20me3, is crucial for activating OCT4 during iPSC reprogramming.^281^ Another intriguing family of anti-ageing drugs that can significantly contribute to the fight against ageing and age-related disorders are histone deacetylase (HDAC) inhibitors. HDAC inhibitors have the ability to reverse the deacetylation of histone tails and trigger the expression of specific genes.^282^

### Rejuvenating strategy by clearance of SCs and decreasing SASP

#### Elimination of SCs using genetic approaches

Recent advances in our understanding of gene manipulation against age and age-related diseases have prompted several distinct interventional strategies. The first one is the repression of senescence-associated genes to delay or reverse senescence. For example, identifying the driving role of the histone acetyltransferase gene KAT7 in senescence in the Werner syndrome (WS) and Hutchinson-Gilford progeria syndrome (HGPS) models via genome-wide CRISPR-Cas9-based screening. Intravenous administration of lentiviral vectors containing Cas9/sg-KAT7 reduced liver ageing and increased lifespan in progeroid and physiologically old mice.^283^ Many genes driving senescence and mediating ageing-related disease have been revealed in the past year, and some resource tools also have been developed to facilitate the identification of genes related to cellular senescence and age, including SeneQuest (available at http://Senequest.net) and GenAge: The Ageing Gene Database (available at https://genomics.senescence.info). These ageing-associated genes provide the therapeutic targets for rejuvenation via gene ablation Then, targeting the inhibition of anti‐apoptotic regulators to increase the susceptibility SCs to apoptosis is another approach to eliminating SCs. Analysis of proteomic and transcriptomic datasets revealed senescent cell antiapoptotic pathways (SCAPs), covering Bcl-2 family members, ephrins, PI3K isoforms, p21CIP1, HIF-1α, and plasminogen-activated inhibitors 1 and 2 (PAI-1 and -2), were indeed more highly expressed in senescent than non-senescent cells, which is responsible for the resistance of SCs to apoptosis.^284^ The small interfering RNAs (siRNAs) against these anti‐apoptotic regulators were effective in killing SCs.^285^ However, more than one SCAP pathway mediate the resistance to apoptosis, so the gene ablation targeting a single SCAP pathway may be poor. Furthermore, the dominant SCAPs also vary with different cell types, which expands the limitations of genetic silence of SCAPs to remove SCs. Genetic strategy against senescence also can be achieved by overexpression of longevity gene to reset gene expression in SCs. Telomerase reverse transcriptase (TERT) and follistatin (FST) gene therapy using high-capacity cytomegalovirus vector by intranasal inhalation or injection significantly improved phenotypes associated with healthy ageing and extended lifespan in mice without severe side effects.^286^ Specifically, glucose tolerance and physical performance were significantly enhanced. Further, TERT reduced the telomere shortening associated with ageing, and both therapies halted the degeneration of the mitochondrial structure.^286^ A clinical trial about TERT gene therapy with adeno-associated virus (AAV) vector to reverse ageing has been approved, and it is also the first clinical trial of genetic editing against ageing that was registered in Clinicaltrials.gov in the world (NCT04133649). Another study reported the gene therapy with three longevity genes (FGF21, sTGF-βR2, and αKlotho) treated multiple age-related diseases.^287^ In obese and diabetic mice, the findings demonstrated that FGF21 alone totally reversed weight gain and type II diabetes, and that when combined with sTGF-R2, renal fibrosis in mice reduced renal atrophy by 75%. Administration of sTGF-βR2 alone or in combination with two other genes improved cardiac function by 58% in mice with heart failure, indicating that the combination of FGF21 and sTGF-βR2 successfully treated all four diseases with improved health and survival. Importantly, the injected gene did not change the animal’s native DNA, remained separate from its natural genome at all times, or transmit between living things or to subsequent generations.^287^ These results demonstrate the potential of single or combination gene therapy to treat ageing and ageing-related diseases.

#### Pharmacological intervention targeting SCs

The association between SCs burden and health span helps to discover chemicals that selectively remove senescent cells. The fact that SCs exhibit resistance to apoptosis supports the idea that these SCs rely on antiapoptotic, pro-survival mechanisms to prevent self-destruction.^288^ Recent advances have evoked the development of senolytic compounds that work to target the SCAP network nodes and impair the defenses of SCs against apoptosis. Based on bioinformatic analysis, forty-six compounds that target SCAP pathways have been identified as possibly senolytic.^284^ Senolytic drugs with established safety profiles or FDA approval for additional human purposes were initially and purposefully chosen for further study in order to speed the transition from the bench to the bedside, such as SRC/tyrosine kinase inhibitor dasatinib (D), natural flavonoids quercetin (Q) and fisetin (F). D has the ability to trigger apoptosis brought on by dependency receptors, like as ephrins, in part through blocking Src kinase.^289^ Senolytic flavonoids primarily induce apoptosis by blocking members of the BCL2 family such BCLxL, as well as HIF-1 and other SCAP network elements.^290^ Because the different contributions of nodes across the SCAP network in SCs subpopulations, senolytic drugs have cell or tissue-specific sensitivity. For example, senescent human pre-adipocytes or MSCs are susceptible to D but not Q or F, while senescent HUVECs are susceptible to Q or F but not D.^284^ Likewise, targeting a single SCAP may not be effective in getting rid of SCs due to the heterogeneity of SCAPs among various types of senescent cells. Either D or Q alone has the inability to eliminate senescent mesenchymal embryonic fibroblasts from Ercc1^−/−^ mice and bone marrow mesenchymal progenitors from old mice, but the combination of these agents can do that.^284^ In vivo experiment revealed that, both in senescent cell-transplanted young mice and naturally aged mice, intermittent oral administration of D + Q alleviated physical dysfunction and reduced mortality hazard to 65%, and meanwhile increased post-treatment survival by 36% while.^291^ Senolytic agents that focus on a single SCAP node tend to trigger apoptosis in a restricted range of SCs types. Navitoclax (ABT-263), a potent antagonist of BCL-2 and BCL-xL, eliminates senescent myocytes and hematopoietic stem cells but not senescent pre-adipocyte.^292^ As specific BCL-xL inhibitors, A1331852 and A1155463 have senolytic activity against HUVECs and IMR90 cells. albeit not senescent human pre-adipocytes.^290^ The discovery of SCAPs promotes the occurrence of more senolytic strategies, and senolytic drugs hold great promise in treating ageing-related diseases.

#### Immune-mediated SC clearance

Despite the apoptotic SCs induced by senolytic strategies, such apoptotic cells are still finally removed by the immune system. NK cells, macrophages, and cytotoxic T cells are innate and adaptive immune cells that carry out immunosurveillance of SCs and play a crucial role in their elimination when they are young or under physiological conditions. The interaction between the activating NKG2D receptor and its ligands expressed on SCs determines the involvement of NK cells in the elimination of SCs. In cycling human endometrium, decidual senescence is required to support embryonic implantation, and acute decidual senescence can promote the release of IL-15 and attract uterine NKs, further selectively targeting and clearing senescent decidual cells through the interaction of NKG2D and DNAM1 receptors-mediated granule exocytosis.^293^ SCs can also be eliminated by tissue-resident macrophages or circulating monocytes (MOs). After delivery, MOs are crucial in removing senescent uterine cells to preserve postpartum uterine activity, which helps to maintain the likelihood that a second pregnancy would be successful.^294^ T cells are also essential players in immune surveillance and healthy longevity. Similar to NKs, CD8 + cytotoxic T cells can function by granular exocytosis and express the same receptor, NKGD2, to identify SCs.^295^ By identifying MHC II molecules, CD4^+^ cytotoxic T lymphocytes can directly destroy senescent tumor cells. Accumulating evidences have demonstrated that SCs could be cleared by immune cells, which could be a lever for lowering the burden of SCs. Future researches need to focus on new approaches, such as the use of chimeric antigen receptors (CAR) or modified T-cell receptors to enhance immune cells’ capacity to recognize and eliminate SCs, or explore various approaches including vaccines and small molecule immunomodulators, to enhance the selectivity and efficiency of immune clearance.

#### Inhibition of SASP

SASP inhibitors, also named senomorphics, target pathways such as p38 MAPK, JAK/STAT pathway, and ILs, further attenuating the SASP of SCs. Suppressing the SASP also has been an alternative strategy for combating cellular senescence-associated phenotypes or diseases. p38MAPK is the MAPK family member and also an important regulator of the SASP, and p38MAPK inhibitors including SB203580, UR-13756, and BIRB 796, potently suppressed SASP expression in SCs.^296^ JAK inhibitors repress SASP function, alleviate systemic inflammation, and eventually enhance physical function.^297^ Glucocorticoids act as potent inhibitors of selected pro-inflammatory cytokines (IL-6, IL-1α, and MCP-1).^298^ As a flavonoid, apigenin (4’,5,7,-trihydroxyflavone) could suppress the level of IL-1α, IL-6, and CXCL10, and attenuate inflammation as well as the related chronic diseases of ageing.^299^ ROCK inhibitor Y-27632 is also recognized as a senomorphic drug, which has the ability to reduce levels of pro-inflammatory cytokines secreted by senescent normal and dysplastic oral keratinocytes but with no effect on the permanent cell growth arrest.^300^ Alternatively, senomorphics may be achieved by specific neutralizing antibodies against individual SASP factors. Of note, unlike the intermittent administration of senolytic drugs, most SASP inhibitors are needed to maintain suppression of SASP in a manner of continuous treatment, which raises the likelihood of side effects compared to senolytics taken on an intermittent schedule.

### Rejuvenating strategies to restore aged stem cell functions

#### Improving metabolism in aged stem cells

The metabolic disturbance is known to drive the function decline of adult stem cells with respect to ageing, and the mechanism by which nutrient-sensitive signaling pathways such as the mTOR and AMPK pathways play a central role.^301^ Targeting nutrient-sensitive signaling pathways to regulate metabolism homeostasis may be the alternative strategy to rejuvenate aged stem cells.

Rapamycin’s suppression of mTORC1 slows the ageing of mouse long-term stem cells (LT-HSCs) by maintaining their ability to self-renew and produce blood cells.^302^ Rapamycin also rescues adult EpSCs exhaustion and the resulting progressive hair loss in mice.^303^ In addition, rapamycin improves MuSCs function through induction of autophagy in the *Ercc1*^*-/Δ*^ progeria mouse model.^304^ In the *Zmpste24*^*-/-*^ progeria mouse model, rapamycin-treated primary MuSCs could restore differentiation and proliferation potential and reverse senescence.^305^ Despite the well-established effects of rapamycin on lifespan, it remains debated whether this drug is rejuvenating compound. Long-term (1 year) rapamycin treatment increased memory and learning in both young and old mice, but it also improved some of these traits in young animals, suggesting that rapamycin has beneficial effects that are age-independent.^306^

Metformin also contributes to metabolic-induced rejuvenation by regulating nutrient-sensitive signaling.^306^ Metformin has been shown to extend longevity and improve health in mice and *C. elegans* by activating AMPK in a LKB1-dependent mechanism.^307^ Metformin also prevents the AGEs-induced inflammatory damage and neuronal dysfunction in human neural progenitor cells through activating AMPK.^308^ Similarly, metformin rejuvenates ageing oligodendrocyte progenitor cells and restores CNS remyelination capacity by activating the AMPK-mediated mechanism.^309^ Metformin is also shown to directly inhibit mTORC1 for improving the stemness and alleviating senescence of stem cells, such as the reversal of ageing-associated characteristics in intestinal stem cells.^310^ Using metformin to improve metabolism for cell rejuvenation and the treatment of age and age-related diseases such as degenerative skeletal diseases is proved to be promising.^311^

#### Targeting DNA repair pathways to rescue aged stem cell functions

To maintain genomic integrity and tissue homeostasis, adult tissue-specific stem and progenitor cells have defensive mechanisms that reduce endogenous DNA damage. However, the DNA repair response in stem cells declines with ageing, which results in the loss of stem cell properties and DNA damage-caused cellular senescence and organ atrophy.^312^ Identifying a suitable target to promote DNA repair may contribute to rescuing the DNA damage-induced function loss in aged stem cells. NAD^+^, as hydride-accepting coenzyme, is involved in the DNA repair and preservation of genome stability, and the increased NAD^+^ level reduces DNA damage.^313^ NAD^+^ can act as a substrate for PARP and SIRTs and provide adenylate for DNA ligase IV, facilitating nuclear DNA repair.^313^ NAD^+^ levels can be increased by supplementation with nicotinamide mononucleotide (NMN), inhibiting NAD^+^-consuming enzymes, and regulating NAD^+^ biosynthetic enzymes, in which NMN is widely used. In telomere-dysfunctional animals, administration of the NMN preserves telomere length, inhibits the DNA damage response and p53, enhances mitochondrial activity, and reverses liver fibrosis.^314^ NMN alleviates retina damage, drops DNA damage by 50%, and eliminates SCs in age-related macular degeneration models.^315^ NMN protects skin cells such as keratinocytes from UV radiation-caused DNA damage by enhancing DNA repair, contributing to reverse skin ageing.^316^

#### Reversing stem cell ageing by improving protein homeostasis

The conducive effect of proteostasis loss on stem cell ageing inspires the strategy of protein homeostasis to protect stem cell function. Chemical/biochemical therapy is an important approach capable of inhibiting the protein aggregation process, and it has received extensive research in “protein misfolding neurodegenerative diseases (PMND)”. Numerous chemical chaperones, including 4-Phenylbutyric Acid (4-PBA) and its derivatives, have been shown to exhibit anti-amyloidogenic activity and to prevent misfolding protein aggregation. By increasing partially folded proteins and stabilizing their more compact native structures, 4-PBA can reduce the formation of unfolded aggregates and increase the pool of folding intermediates. It can also change the structure of Hsps to increase the exposure of hydrophobic surfaces, which improves Hsp chaperone activities.^317^ In the face of misfolding-induced stress, 4-PBA can elevate the proteostasis ability via activating the heat shock response (HSR) and UPR^mt^ pathway.^318^ As dual-targeting drugs, chalcone-based derivatives, including nordihydroguaiaretic acid (NDGA), curcumin, resveratrol, quercetin, and isoliquiritigenin, can target both amyloid aggregations and the proteostasis network in vivo.^319^ The mechanism is partially linked to Hsps-containing protein complexes and ER stress-mediated cellular activity.

#### Restoring the stem cell pools in ageing tissues

The depletion of adult stem cell pool with age contributes to tissue degeneration, so maintaining tissue homeostasis into old age requires replenishing the stem cell pools. Stem cell transplantation is a therapeutic intervention, and it has been extensively researched as a way for the replenishment of regenerative cells to provide stem cells from an unaffected donor or gene-corrected autologous cells to the recipient. A large number of adult stem cells are available for autograft or allograft therapy, but only HSCs transplantation is widely accepted and used clinically, which has been demonstrated to be successful in treating hematologic disorders such as leukemia and lymphoma.^320^ Animal experiments revealed replenishing the stem cell pools with stem cell transplantation is also possible for other tissue. For example, EpSCs transplantation can regulate inflammation response, remodel the microenvironment of skin wounds, recapitulate tissue integrity and promote diabetic wound healing.^321^ MuSCs have the ability to facilitate myofibers regeneration, restore dystrophin expression, and markedly improve contractile function. More importantly, transplanted MuSCs also accessed the satellite cell compartment, replenishing the endogenous stem cell pool and taking part in injury repair.^322^ Of note, considering the susceptibility of stem cells to ageing, simple cellular replacement therapy with younger stem cells is insufficient. It may be worthwhile to pursue methods for reactivating residual stem cells or combining such procedures with stem cell transplantation. For instance, inhibition of the p38 MAPK signaling pathway in endogenous MuSCs can rescue muscle regeneration ability in ageing animals.^323^ Triboelectric stimulation boosts the pluripotency and differentiation potential of old BMSCs, enhances their proliferation and reverses senescence phenotypes. The mechanism by which is in a manner of MDM2-dependent p53 degradation.^324^ Therefore, triboelectric stimulation can be used to rejuvenate endogenously aged BMSCs and replenish the bone stem cell pool, further resulting in the acceleration of bone repair in elderly patients.^325^ In addition to adult stem cells, reprogramming-derived stem cells can act as alternative cell sources for transplants. A recent clinical trial reported implantation of iPSC-derived dopaminergic progenitors into the putamen (left hemisphere followed by the right hemisphere) of a patient with PD indeed improved clinical symptoms of PD after surgery.^326^ Reprogramming-derived stem cells are highly amenable to genetic correction and homogeneous than isolated adult stem cells, but there still exist major obstacles limiting the application, including low reprogramming efficacy and potential security concern.

### Rejuvenating tissue-specific cells by targeting their microenvironment

#### Targeting extracellular signals to reverse stem cell ageing

Physiologically, extracellular signaling molecules are cues, such as EVs, neurotransmitters, growth factors, hormones, and cytokines, designed to transmit specific information to target somatic cells or adult stem cells. EVs function as a cutting-edge tool for stem cell rejuvenation because of their ability to transfer genes safely and with systemic effects. For instance, ESC-EVs can directly facilitate pressure ulcer repair in ageing mice through the rejuvenation of tissue-resided senescent endothelial cells.^327^ Neonatal MSCs-secreted EVs can deposit PCNA to reverse aged BMSCs and slow age-related degeneration.^328^ In addition, neurotransmitters have been reported to regulate the behavior of nerve-dependent tissue stem cells in wound healing.^329^ Lgr6^+^ EpSCs are a regionally restricted population of EpSCs that has the ability to generate all skin lineages and interact with nerves and specialize in wound re-epithelialization, and the activation of Lgr6^+^ EpSCs rely on neurotransmitters from neuroendocrine signaling and subsequently, promotes wound repair.^330^ These results suggested that targeting the activation of neuroendocrine signaling might be a promising approach to facilitate wound healing.

#### Rejuvenating aged cells by environmental preconditioning

Injured tissue has a hostile environment, including inflammation, immune impairment, hypoxic stress, and poor blood supply, which degrades stem cell function, promotes cellular senescence, and results in a low survival rate of transplanted stem cells in vivo. Therefore, it is essential for stem cells to remain viable and maintain their potency before inducing a strong repair response. The optimization of the culture environment, such as hypoxic pretreatment, may achieve preconditioning-induced protection for stem cells.^331^ The oxygen tensions in natural cell microenvironments appear to be substantially lower. Particularly, ESCs require low oxygen levels to survive, which start at embryonic implantation and last throughout fetal development. The absence of maternal circulation causes a hypoxic environment during embryo implantation, and in the early stages of pregnancy, the oxygen content of the uterine surface is typically only 2%.^332^ Placental oxygen levels only rise to about 8% even after the embryo forms the connection to the maternal vascular system.^333^ Relative hypoxia is a feature of the normal physiologic environment of ESCs, and thus, low oxygen concentrations enhance the more efficient growth of ESCs. Adult stem cells such as HSCs and BMSCs, similarly live in hypoxic conditions in vivo.^334^ For these reasons, hypoxic pretreatment in vitro promotes the long-term expansion of stem cells, delays replicative senescence, and improves the therapeutic potential.

#### Modulating ECMs for maintaining tissue homeostasis

The extracellular microenvironment’s chemical and mechanical characteristics are sensed by tissue-specific cells, which then produce responses that control a variety of cellular processes, such as expansion, migration, and differentiation, and activation, further maintaining tissue homeostasis. For example, integrin receptors-mediated cell adhesion to the ECM is necessary for cell cycle progression, particularly during G2 to M transition and early mitosis.^335^ In addition, the structural integrity of the ECM and integrin-associated proteins may also contribute to cellular energy metabolism, because the extracellular matrix proteins regulate nutrient and hormonal flux to balance the energy uptake.^336^ Changes in the biophysical characteristics of tissue affect how mechanical impulses are received by cells in a variety of disease states, inflammatory conditions, and ageing. Through the process of mechano-transduction, these mechanical cues are transformed into biochemical signals that affect immune cell functions such as cell activation, cytokine generation, metabolism, proliferation, and trafficking.^337^ As an integral component of all organs, the alternations of biomechanical and biochemical cues exhibit a huge effect on cell behavior and tissue homeostasis. ECM molecules are continuously produced and secreted throughout life, in both physiologically healthy and pathological states, and they regulate a wide range of biological functions, including stem cell differentiation, angiogenesis, innervation, and wound healing.^338^ Therefore, the modulation of ECM to induce desirable cell-specific responses for cellular rejuvenation is of great importance to tissue homeostasis. Because ECM has a more favorable pro-remodeling host immune response and can offer a natural, instructional microenvironmental habitat for functional tissue remodeling, ECM-based biomaterials have been emerging and exhibit great variability.

#### Restoring defective intercellular communications to extend healthy lifespan

Intercellular communication has double-edged–sword activities, contributing to tissue homeostasis maintenance but also detrimental in ageing and related diseases, and altered intercellular communication has been the hallmark of ageing.^7,77^ One of the most prominent and important changes in intercellular signaling that occurs with age is “inflammageing”. The dominant role of inflammation in ageing-related intercellular communication raises the potential of anti-inflammatory agents in lifespan. Aspirin is a common example because it can prolong mouse life and promote good ageing in humans.^339^ Moreover, age-related changes in the transcriptional landscape of tissues have highlighted the importance of NF-κB-induced inflammatory responses, and the targeted inhibition of this transcription factor may restore the altered intercellular communications for rejuvenation. The conditional expression of an NF-κB inhibitor in transgenic mice’s aged skin rejuvenates the tissue’s phenotype and restores the transcriptional signature associated with youth.^340^ The novel relationship between inflammation and ageing is that NF-κB-mediated hypothalamic immunity causes neurons to produce less gonadotropin-releasing hormone (GnRH), and this decrease in GnRH can be a factor in several age-related changes, including decreased neurogenesis, muscle weakness, skin atrophy, and bone fragility. Correspondingly, GnRH treatment rejuvenates ageing-impaired neurogenesis and prevents ageing development.^341^

### Restoration of proper circadian clock for anti-ageing

#### Rejuvenation of the circadian clock by dietary restriction

The use of DR, including time-restricted feeding (TRF) and calorie restriction (CR), has many profound beneficial effects on ageing through the regulation of circadian clock.^342^ TRF, also known as limiting the time and length of food availability with no calorie decrease, can trigger clock-associated gene expression to drive rhythms in circadian mutant mice. For example, the transcripts, metabolites, and proteins in WT liver exhibit daily rhythms, whose rhythms are lost in clock gene knockout (CRY1^-^/CRY2^-^; PER1^-^/PER2^-^; BMAL1^-^) mice, while TRF partially rescues the disturbed rhythms in these mutant mice.^343^ In addition to the liver, TRF modifies the phase and amplitude of the skin’s circadian clock to rejuvenate ultraviolet radiation-caused skin ageing in old animals.^344^ For lifespan, TRF even drives a clock-dependent increase in autophagy activity to induce longevity extension.^345^ CR, a dietary regimen low in energy without malnutrition, can decrease the occurrence of age-related diseases and extends the lifespan via controlling circadian clock. It has been reported that the duration of two months for CR intervention in early life could enhance the amplitude of core clocks in liver.^346^ From the regulatory site, the central oscillator is impacted by CR, which also entrains the SCN clock. The SCN clockwork’s temporal organization, circadian outputs under the light-dark cycle, and circadian system photic responses are all influenced by CR during the daytime.^347^ BMAL1-dependent and BMAL1-independent pathways are the primary transcriptional and post-transcriptional mechanisms by which CR modifies the clocks. A functional circadian clock system is necessary for CR-mediated lifespan extension, as evidenced by the fact that CR fails to extend the lifespan of BMAL1 knockout mice.^348^ Comprehensively, the circadian clock system extends longevity through transmitting the anti-ageing signals brought on by DR.

#### Genetic regulation of the circadian clock rescues an aged phenotype

Circadian clock-associated genes modulate the extrinsic and intrinsic mechanisms in lifespan modulation and organ ageing. The circadian clock in intervertebral discs (IVDs) is functional and temperature-entrainable, and ageing disrupts the circadian rhythm of IVDs in an inflammation-dependent manner and leads to IDD.^349^ Restoring the dampened expression of BMAL1 protects against IDD via inhibiting RhoA/ROCK signals.^350^ The expression of CLOCK is increased in OA cartilage, and by reestablishing autophagic flow, ablation of CLOCK increases antioxidant enzyme activity, lowers ROS generation, and alleviates chondrocyte senescence.^120^ The downstream gene of CLOCK-BMAL1 complex CRY2 is suppressed in mice with ageing-related OA, which makes the loss of ECM homeostasis in cartilage.^351^ It also reported the low mRNA and protein expression level of CRY in the old Drosophila melanogaster. The mRNA oscillatory amplitude of multiple genes involved in the clock mechanism was dramatically increased in elderly flies by restoring CRY using the binary GAL4/UAS system, and the flies with higher CRY also displayed a remarkable extension of health span.^352^

#### Pharmacological activation of circadian clock for anti-ageing

Numerous parts of human physiology are regulated by the circadian rhythm, opening up windows for interventions that can be made by only giving medications when their targets are at the proper expression level to rescue. There is growing interest in developing small molecules that directly target the circadian system for medicinal benefits. Representative chemicals targeting core clock components include KL001, SR9009/SR9011, and Nobiletin. KL001 specifically interacts with CRY, which prevents ubiquitin-dependent degradation of CRY, leading to extending circadian time.^353^ KL001 suppressed glucagon-caused gluconeogenesis in primary hepatocytes and improved glucose tolerance in obese mice.^353^ KL001 also extends the lifespan and modifies the circadian rhythms of Drosophila melanogaster.^354^ The metabolic regulators SR9009 and SR9011 pharmacologically target REV-ERB receptors. By lowering fat mass and significantly enhancing dyslipidaemia and hyperglycemia, SR9009/SR9011 can change the circadian pattern of expression of a variety of metabolic genes to enhance energy expenditure and lower obesity.^355^ Nobiletin, a natural polymethoxylated flavone targeting agonizing retinoid acid receptor-related orphan receptors (RORs), is a clock amplitude-enhancing small molecule that has the ability to directly engage circadian cellular clocks to support metabolic health in illness models and healthy ageing in naturally ageing mice.^356^ Nobiletin is capable of ameliorating steatosis in obesity by restoring aberrant hepatic circadian rhythm,^357^ and alleviating cognitive deficits as well as the pathological aspects of AD, such as Aβ pathology, hyperphosphorylation of tau, astrogliosis-associated neuroinflammation, and oxidative stress.^358^ There are other small-molecule modulators with the function to regulate circadian rhythm, which do not directly target core clock genes, such as CKI-7 (CK1 inhibitor), Lithium (GSK3β inhibitor), and OPC-21268 (antagonists for arginine vasopressin receptors V1a).^359^ The role of these chemical regulators of circadian clock in the rejuvenation of ageing or ageing-related diseases remains being explored.

### Organismal rejuvenation via immune system-targeting therapeutic approaches

#### Therapeutic targeting of immune cells

Immune system is interconnected with all the other systems in the body, and this systemic nature provides the potential opportunity that targeted modifications to a small group of cells (e.g., HSCs or T lymphocytes) for improving the health of various organ systems.

As the origin of blood cell lineages, HSCs are multipotent precursors population that can reconstitute the hematopoietic system and sustain immune homeostasis. Ageing HSCs have less self-renewal activity, fewer cell divisions, decreased homing efficiency and myeloid lineage-biased differentiation as well as reduced output of lymphoid progenitors.^360^ HSCs play a crucial role in the immune system, hence functional restoration of ageing HSCs has great significance to immune rejuvenation and organismal health. HSCs ageing is accompanied by alterations at the gene level, and genetic modulators by ablation of genes driving ageing or overexpression of rejuvenative genes might be strategies to prevent HSCs dysfunction, such as the deletion of the imprinted gene Grb10 or forced expression of Satb1.^90^ In addition, pharmacological intervention based on the pathophysiology of HSCs senescence also can promote rejuvenation of the HSC compartment, reconstitute the immune system activity and finally even increase overall lifespan. For example, supplementing elderly mice with a sympathomimetic that specifically targets the adrenoreceptor β3 (β3-AR agonist, BRL37344) greatly improved the in vivo function of aged HSCs.^361^ In progeroid mice, BRL37344 can decrease premature myeloid and restore the proximal association of HSCs to megakaryocytes.^362^

Immune lineage-mediated cellular rejuvenation mainly focuses on the restoration of T lymphocytes exhaustion caused by organismal ageing, due to its high susceptibility to ageing-associated changes to the immune system.^363^ Adoptive transfer of progenitor T (proT)-cells is the merging approach to facilitate T cells function recovery. Preclinical results have shown that proT cells could effectively engraft involuted ageing thymuses, achieve rapid long-term thymic reconstitution and accelerate T cell recovery.^364^ The process is regulated by receptor activator of NF-B (RANK) and receptor activator of NF-B-ligand (RANKL) interactions, which generate chemokine-rich niches within the cortex and medulla that probably encourage the recruitment of bone marrow-derived thymus seeding progenitors.^365^ ProT cells are limited to the recipient’s major histocompatibility complex (MHC) and produce host-tolerant T lymphocytes after undergoing positive and negative selection in the host thymus. Therefore, ProT cells avoid the clinical concerns brought on by graft-versus-host disease (GVHD), and it provides an alternative cell-based strategy to rejuvenate T-cell immunity clinically. Surprisingly, immunotherapy with T cell activation, such as the blockage of PD-1/PD-L1, provides a novel alternative to rejuvenate ageing phenotypes.^366^ In SCs, PD-1/PD-L1 axis inhibition could activate CD8^+^ T cells and improve senescence surveillance, further resulting in the reduced number of p16^+^ cells. Besides, anti-PD-1/PD-L1 administration also ameliorated age-associated function decline in vivo, including decreased SASPs and inflammation, and improved alveolar volume, fat accumulation in the liver, grip strength, and athletic ability.^366^ Clinically, immune checkpoint blockade (ICB) has been widely used, and thus, ICB against ageing is promising.

#### Other immune-targeting strategies

As the key site of T lymphopoiesis, the thymus orchestrates adaptive immune responses, and ageing-associated thymic atrophy or involution contributes to adaptive immune system deviations. Pharmacologic interventions to increase thymopoiesis have been reported, such as IL‐21, which exhibits beneficial effects on thymic function recovery and T cell output in the aged models.^367^ The effect is systemic but is also transient and limited. Cellular reprogramming provides an alternative avenue for thymus function restoration and immune rejuvenation. Chemical activation of thymus organogenesis program can direct human ESCs to differentiate into thymic precursor lineage, further promoting functional regeneration of human thymus in vivo.^368^ First treatment with activin A directs the fate of hESCs towards the definitive endoderm, and the subsequent regulation in signaling pathways of Wnt, TGF‐*β*, Shh, retinoic acid, BMP4 and FGF facilitates the generation of thymic epithelial progenitors (TEPs).^368^ hESCs-derived TEPs could yield functional thymic epithelial cells when engrafted into athymic mice. T cells produced in TEPs-recipient mice have functional properties capable of in vitro expansion and in vivo immune responses, and they were detected 10 weeks post-transplantation in the peripheral blood. However, the regenerative efficiency of hESCs-TEPs generated by these protocols is low and there is a progressive decrease in the quantity of T cells produced, which was not sustained over 22 weeks.^368^ Thymocyte lineage-specific gene FOXN1 manipulation can drive thymus regeneration and restore its function in the age murine.^369^ iPSCs also can be directed towards thymus fate by ectopic expression of FOXN1.^370^ FOXN1-mediated iPSC-TEPs contribute to supporting T cell development, and antigen stimulation can cause the produced T cell to activate, which reveals that FOXN1 can promote the functional regeneration of human thymus from iPSCs.^370^ Similarly, FOXN1 mediates ectopic thymus regeneration from fibroblasts, and the fibroblasts-derived thymus also can revitalize the thymic architecture overall, boost the number of naive T cells, and decrease the number of senescent T cells and inflammatory.^371^

### Targeting systemic signals for organismal youthful state

#### Heterochronic transplantation and parabiosis for regeneration

In 2005, heterochronic parabiosis was initially demonstrated to reverse the age-dependent loss in stem cell function by restoring the proliferation and regeneration capacity of aged satellite cells and hepatic progenitor cells in naturally aged mice.^372^ Since then, heterochronic parabiosis has been used as an experimental system to examine potential systemic influences on lifespan, age-related diseases, and the process of organismal ageing. For example, in the central nervous system, heterochronic parabiosis showed young mice exposed to an old systemic environment or plasma from old mice exhibited diminished synaptic plasticity, and worse contextual fear conditioning and spatial learning and memory, while systemic infusion of young blood plasma reversed that process.^373^ It also revealed that plasma levels of chemokines—including CCL11 were linked to decreased neurogenesis in aged mice. Young mice with elevated peripheral CCL11 chemokine levels in vivo suffered adult neurogenesis loss as well as learning and memory impairments., which provided a new therapeutic target to reverse ageing-caused precipitous decline in neurogenesis.^374^ Similarly, experiments using heterochronic parabiosis also identify another circulating factor, named β2-microglobulin (B2M), which damages adult hippocampal regeneration function and cognitive performance in an ageing-dependent manner. Aged mice and young heterochronic parabionts have high levels of B2M in their blood and hippocampus, and the ablation of endogenous B2M can alleviate age-related cognitive loss and improve neurogenesis.^375^ In addition, heterochronic parabiosis discovered the potential therapeutic value of GDF11 in age-related cardiac hypertrophy and age-dependent deterioration of the neurogenic niche,^376,377^ and the contribution of Ly6G+ plasma extracellular vesicle to improving fracture healing in the elder.^378^ Heterochronic parabiosis has been a valuable experimental system for addressing some of the fundamental issues surrounding the systemic regulation of cell and tissue ageing, which provide corresponding therapeutic opportunities for organismal rejuvenation. Nevertheless, the key obstacles of parabiotic experimentation facing include the difficulty to control experimental procedures, the influences of shared organs, uncontrollable exercise, and an ambiguous blood-sharing onset.

#### Blood factors to revitalize aged organs and tissues

The persuasive evidence that blood factors affect organismal ageing has been provided by heterochronic blood exchange (HBE), which has shown that circulating factors not only restore youthful traits to aged tissues but also cause systemic senescence in the young organism. Thus, defining the blood mediators of the rejuvenating effects is of great importance to revitalize aged organs and tissues. The deep proteomic analysis from the large-scale population has confirmed many increased plasma proteins with ageing, like sclerostin, pleiotrophin, and transgelin, while epidermal growth factor receptor (ERBB1) and a2‐antiplasmin were decreased with age.^379^ Quantification of proteomic alternations revealed ageing protein waves across the lifespan, and for example, structural pathways such as the ECM are downregulated at a young age but increased in middle and old age.^379^ Metabolomic analysis has identified the blood metabolic profiles that change with ageing or ageing-relevant conditions. A nontargeted, quantitative metabolomics analysis in the blood of 15 young and 15 elderly individuals reported that 14 metabolites showed significantly remarkable age-related alternations.^380^ The increasing metabolites comprised citrulline, pantothenate and N6-acetyl-lysine, and were associated with declining renal and liver function, while the decreasing counterparts included NAD^+^, nicotinamide adenine dinucleotide phosphate (NADP^+^) and UDP-acetyl-glucosamine, which mainly were associated with redox homeostasis. A longitudinal examination of the impacts of ageing on the blood plasma metabolome also identified pathways enriched for age-related metabolites embodied tryptophan, nucleotide, and xenobiotic metabolism.^381^ HBE also gives clues to the role of circulating EVs in ageing rejuvenation. It was reported that aged mice exposed to young blood showed muscle regeneration, in which serum EVs containing α-Klotho play a key role, and administration of Klotho-enriched EVs into ageing mice could promote muscle regeneration.^382^ Specifically, identifying blood factors that promote or antagonize ageing can provide therapeutic opportunities for systemic rejuvenation. For example, dilution of old blood plasma with saline that contained 5% albumin to create a “neutral” age blood exchange (NBE), which met or exceeded the rejuvenating effects of promoting muscle repair, lowering liver adiposity and fibrosis, reducing neuroinflammation, and boosting hippocampal neurogenesis in elderly mice.^383^ These results suggested that blood factors might be the key players in ageing process, and targeting blood factors is expected to yield robust resetting of the systemic signaling to youth.

## The importance of cellular rejuvenation in treating human diseases

The accumulation of SCs during ageing accelerates the onset of ageing‐related disorders and chronic diseases, including metabolic disorders, degenerative disorders, inflammation, autoimmune diseases, cancers, etc. Deeper insights into the role of cellular senescence in disease pathogenesis highlight the significance of cellular rejuvenation in treating human disorders, and also provides cues for rejuvenation therapies (Table 1). In addition, a variety of pharmacological treatments with the elimination of SCs or reversal of tissue and organ ageing have been developed for cellular rejuvenation (Table 2). The discussion that follows will thus concentrate on the advances in rejuvenation strategies for alleviating human diseases, and the details of clinical trials in this study were shown in Table 3.

**Table 1:** The cellular rejuvenation used for various disease treatments

Rejuvenation strategy	Specific intervention	Mechanism	Disease treatment	Ref
Cellular reprogramming	Reprogramming to a fully pluripotent state	1. Reprogramming from cancer cells to iPSCs with a benign phenotype 2. iPSCs modeling of diseases 3. The transplantation of iPSCs-derived functional cell	Cancer, AMD, AD, PD, ALS, diabetes, OA, osteoporosis and autoimmune disorders	^326,473–480^
	Lineage reprogramming	1. Cell replacement therapy of reprogramming-derived functional cells without the need for a pluripotent state 2. Reprogramming cancer cells to differentiated cells to alleviate tumorigenicity	Cancer, diabetes, AD, PD, CAD and fibrosis	^438,481–485^
	In vivo reprogramming	1. In vivo partial reprogramming (transient expression of pluripotency-associated genes) for epigenetic rejuvenation and the amelioration of organismal phenotype associated with ageing 2. In situ trans-differentiation of targeted cells to functional cells	Ageing, diabetes, tissue regeneration, autoimmune disorders, CAD and fibrosis	^272,419,486–488^
SCs elimination and SASPs inhibition	Ablation of p16-positive cells	Pharmacological activation of transgene INK-ATTAC to induce apoptosis in p16(Ink4a)-expressing cells	Age-related functional decline of kidney, heart, cartilage and bone	^489,490^
	Senolytics	The targeted inhibition of the SCAP network to induce SCs apoptosis (e.g., Dasatinib, Quercetin, Fisetin and Navitoclax)	Cancer, diabetes, ageing, fibrosis, osteoporosis, obesity, NDD, CAD, IVDD and CKD	^491–495^
	Senomorphics	Suppressing the signaling from SCs and reducing systematic inflammation (e.g., p38MAPK inhibitor, JAK inhibitors, rapamycin, glucocorticoids and ILs mAb)	Cancer, diabetes, obesity, NDD, CKD, OA and fibrosis	^406,496,497^
	Immune clearance	Enhancing the function of immune cell to recognize and kill SCs (e.g., NK cells, MOs and T cells)	Cancer, fibrosis and tissue regeneration	^294,498^
Stem cells-associated therapy	Restoration of aged stem cell functions	1. Rescuing metabolism homeostasis (e.g., rapamycin, metformin and NMN) 2. Promoting DNA repair and preservation of genome stability (e.g., NMN) 3. Repairing tissue-specific stem cell to replenish the stem cell pool (e.g., HSCs and EpSCs) 4. Regulating extracellular signals (e.g., EVs delivery and neuroendocrine activation) and remodeling ECMs	Hematological disease, ageing, diabetes, ischemia-reperfusion injury, heart failure, AD and retinal degeneration	^499,500^
	Transplantation of youthful stem cells	1. The differentiation into tissue-specific cell with the stimulation of specific signals and local microenvironment 2. Paracrine effect with the release of soluble cytokines, chemokines and growth factors involved in immune regulation, angiogenesis, anti-apoptosis, anti-inflammation, anti-oxidation and anti-fibrosis	Hematological disease, graft-versus-host disease, organ transplantation, diabetes, inflammatory diseases, and diseases in the liver, kidney, and lung, as well as cardiovascular, bone and cartilage, neurological, and autoimmune diseases	^501^
Dietary restriction	Time-restricted feeding and calorie restriction	1. Nutrient-mediated mechanisms: metabolic regulators, nutritive metabolism pathways, epigenetic mechanisms and circadian clocks 2. Diet-responsive effectors: the diet-endocrine axis, the diet-immune axis, the diet-gut axis, the diet-senescence axis and the diet-nerve axis.	Metabolic syndrome, CVD, Intestinal malfunction, CKD, NDD, MTB infection, COPD, cancer and tissue regeneration	^502^
Immune rejuvenation	Activation of immune cells	1. Restoration of aging HSC to increase the number of immune lineages (e.g., myeloid and lymphoid precursors) 2. Rescuing T lymphocytes exhaustion for the reversal of immune-senescence	Cancer, ageing, age-related degeneration of the thymus, RA and tissue regeneration	^503,504^
	Recapitulation of immune organ	1. Direct differentiation of hESCs/iPSCs into thymic precursor lineage 2. Ectopic thymus regeneration for fibroblasts reprogramming 3. Adult stem cell-derived thymus organoids	Diseases associated with immunodeficiency and autoimmunity	^505^
Heterochronic transplantation	Parabiosis	Allowing the young and old organisms to share of the blood circulation surgically	Ageing, age-related functional impairment of skeletal muscle, liver and bone, cognization decline	^372,377,506^
	Blood or cerebrospinal fluid exchange	1. The delivery of rejuvenative factors by young blood injection, human umbilical cord plasma and young cerebrospinal fluid administration 2. The exchange of half old blood plasma with saline-albumin to dilute age-elevated systemic factors	Ageing, age-related impairment in cognitive function and AD	^383,507^
	Circulating plasma factors	Rejuvenative factors identified by heterochronic transplantation (e.g., GDF11, Apelin, Cadherin13, eNAMPT, SPARCL1, THBS4, TIMP2, Oxytocin, FGF17 and α-Klotho)	Ageing, age-related impairment in cognitive function, skeletal muscle ageing	^508–510^

*HSCs* hematopoietic stem cells, *EpSC* epidermal stem cell, *MDSC* muscle-derived stem cell, *NMN* nicotinamide mononucleotide, *IVDD* Intervertebral disc degeneration, *NDD* Neurodegenerative disease, *CKD* Chronic kidney diseases, *MTB* pulmonary mycobacterium tuberculosis, *COPD* chronic obstructive pulmonary disease, *RA* rheumatoid arthritis, *AD* Alzheimer’s disease, *GDF11* circulating growth differentiation factor 11, *TIMP2* tissue inhibitor of metalloproteinases 2, *FGF17* fibroblasts growth factors 17

**Table 2:** Applications of pharmacological treatment for cellular rejuvenation

Drugs	Pharmacological characteristics	Cellular or tissue target	Rejuvenation on function (examples)	Ref.
Procyanidin C1	Flavonoid	SCs	PCC1 appears to inhibit SASP formation at low concentrations and selectively kills senescent cells at higher concentrations, possibly by promoting production of ROS and mitochondrial dysfunction. Intermittent administration of PCC1 to either irradiated, senescent cell-implanted or naturally aged old mice alleviates physical dysfunction and prolongs survival.	^511^
BPTES	GLS1 inhibitor	SCs	Selective removal of senescent cells via glutaminolysis inhibition and improving age-related tissue dysfunction. Moreover, GLS1 inhibitors can alleviate symptoms of obese diabetes, arteriosclerosis, and nonalcoholic steatohepatitis (NASH) in elderly mice.	^512^
P5091	USP7 inhibitor	SCs	The promotion of the human homolog of MDM2 ubiquitination and degradation by the ubiquitin-proteasome system, further increasing p53 levels and inhibiting the interaction of BCL-XL and BAK to selectively induce apoptosis in SCs.	^513^
Gallic acid	ROS inhibitor	Premature ageing	Gallic acid is a geroprotective compound that has beneficial effects against WS hMSC aging, in which the mechanism containing enhanced antioxidant response, elevated DNA repair ability, improved cell cycle kinetics and inhibited NF-κB pro-inflammatory pathway.	^514^
Rapamycin	mTOR inhibitor	Hematopoietic ageing	Rapamycin promotes ex vivo expansion and long-term hematopoietic reconstitution of HSCs. The increase in long-term hematopoiesis of expanded HSCs is likely attributable in part to rapamycin-mediated inhibition of HSCs senescence by the upregulation of Bmi1 and downregulation of p16.	^499^
Metformin	AMPK agonist	Brain ageing	Fasting or treatment with metformin can reverse the impaired metabolic function and increased DNA damage and restore the regenerative capacity of aged OPCs, improving remyelination in aged animals following focal demyelination.	^309^
Urolithin A	Natural postbiotic compound	Cartilage ageing	Urolithin A improves mitophagy and mitochondrial respiration in primary chondrocytes, and reduces disease progression in a mouse model of OA, as well as decreasing cartilage degeneration, synovial inflammation and pain.	^515^
UK-5099	Hair follicles	Hair follicles ageing	The promotion of lactate production via blocking the entry of pyruvate into mitochondria to activate HFSCs and induce the hair cycle.	^516^
Melatonin	Indole heterocyclic compounds	Ovarian ageing	Melatonin delays ovarian aging by multiple mechanisms including antioxidant action, maintaining telomeres, stimulating SIRT expression and ribosome function, and by reducing autophagy.	^517^
Resveratrol	Polyphenol antioxidant	Cardiac ageing	Resveratrol can exert an adaptive stress response and induce autophagy, forcing the expression of cardioprotective genes and proteins such as heat shock and antioxidant proteins,	^518^
Nicotinamide	NAD (+) ADP-ribosyltransferase inhibitor	Vascular ageing	NMN supplementation increase SIRT1 activity, improves endothelial function and reverses age-related vascular dysfunction by decreasing oxidative stress.	^519^
Y27632	Rock inhibitor	Skin ageing	Y27632 increases COL17A1 expression and regulates epidermal stem cell competition, resulting in the orchestration of skin homeostasis and reversal of skin ageing.	^450^
SGI-1072	DNMT inhibitor	Renal ageing	SGI-1072 contributes significantly to epigenetic renal aging via derepressing NRF2/Klotho pathway.	^520^
BGE-175	PTGDR antagonists	Immune ageing	BGE-175 inhibits the crosstalk between PGD2 and its receptor and rejuvenate immune system in the old, which shows increased migration of dendritic cells from the lungs to the lymph nodes, decreased neutrophil levels in lung tissue, and greatly improved overall survival of COVID models.	^521^
Ginsenoside Rb1	Ingredients of Panax ginseng	Intestinal ageing	GRb1 could improve the intestinal aging via regulating the expression of Sirtuins family and modulating the gut microbiota in the old models.	^522^
Ginsenoside Rd	Ingredients of Panax ginseng	Skeletal muscle ageing	GRd ameliorates aging- and cancer-induced muscle wasting via the inhibition of STAT3 nuclear translocation, scavenging ROS and improving mitochondrial integrity.	^523^

*PCC1* procyanidin C1, *SCs* senescent cells, *GLS1* glutaminase 1, *USP7* ubiquitin-specific peptidase 7, *NASH* nonalcoholic steatohepatitis, *MDM2* mouse double minute 2, *MPC* mitochondrial pyruvate carrier, *DNMT* DNA methyltransferase, *HSCs* hematopoietic stem cells, *HFSCs* Hair follicle stem cells, *OPCs* oligodendrocyte progenitor cells, *PGD2* prostaglandin D2, *OA* osteoarthritis

**Table 3:** Clinical trials of developed therapy in ageing and ageing-related diseases

Treatment category	Intervention	Drug target	Drug dose	Clinical application	Phase	NCT number	Recruitment status
Gene therapy	AAV-hTERT	TERT	Unknown	Ageing	Phase 1	NCT04133649	Recruiting
Senolytic agents	D + Q	D: bcr/abl, src; Q: sirtuins	D: 100 mg; Q: 1000 mg	OS	Phase 2	NCT04313634	Recruiting
	D + Q	D: bcr/abl, src; Q: sirtuins	D: 100 mg; Q: 1000 mg	CKD	Phase 2	NCT02848131	Enrolling by invitation
	UBX0101	MDM2/p53	Unknown	OA	Phase 1	NCT03513016	Completed
	Fisetin	Sirtuins	100 mg	OA	Phase 1/2	NCT04210986	Active, not recruiting
Stem cell therapy	AD-MSCs	Unknown	2 10e8 cells	AD	Phase 1/2	NCT03117738	Completed
	AD-MSCs	Unknown	Unknown	CKD	Phase 1	NCT02266394	Completed
	AD-MSCs	Unknown	Unknown	CKD	Phase 1	NCT01840540	Completed
	BMSCs	Unknown	2 × 10^6^ cells/kg	CKD	Phase 1	NCT02166489	Completed
	BMSCs	Unknown	2 × 10^6^ cells/kg	CKD	Phase 1	NCT02195323	Completed
	UC-MSCs	Unknown	1 × 10^9^ cells	AD	Phase 1	NCT04040348	Active, not recruiting
	UC-MSCs	Unknown	2 × 10^8^ cells	AD	Phase 1/2	NCT02672306	Active, not recruiting
	CNS10-NPC-GDNF	Unknown	5.25 × 10^6^ cells 1.05 × 10^7^ cells	ALS	Phase 1	NCT05306457	Recruiting
Small molecule agents	GSK3008348	αvβ6 integrin	1000 μg	IPF	Phase 1	NCT03069989	Terminated
	PLN-74809	αvβ6 and αvβ1 integrin	Unknown	IPF	Phase 2	NCT04072315	Completed
	IDL-2965	αvβ1, αvβ3, and αvβ6 integrin	Unknown	IPF	Phase 1	NCT03949530	Terminated
	Cenicriviroc	CCR2/CCR5	150 mg	NSLF	Phase 2	NCT02217475	Completed
			150 mg	NSLF	Phase 3	NCT03028740	Terminated
			150 mg	NSLF	Phase 2	NCT03059446	Terminated
			150 mg	NSLF	Phase 2	NCT02330549	Completed
			150 mg	Obesity	Phase 2	NCT02330549	Completed
	Rilonacept	IL-1 receptor	320 mg, 160 mg	SS	Phase 1/2	NCT01538719	Completed
Monoclonal antibody	Tocilizumab	IL-6 receptor	8 mg/kg	OA	Phase 3	NCT02477059	Completed
			162 mg	SS	Phase 3	NCT02453256	Completed
	QAX576	IL-13	10 mg/kg	IPF	Phase 2	NCT01266135	Terminated
			Unknown	Keloid	Phase 2	NCT00987545	Terminated
			Unknown	PFSSS	Phase 2	NCT00581997	Terminated
	Lebrikizumab	IL-13	250 mg	IPF	Phase 2	NCT01872689	Completed
	Tralokinumab	IL-13	400 mg, 800 mg	IPF	Phase 2	NCT01629667	Terminated
	SAR156597	IL-4/IL-13	200 mg	SS	Phase 2	NCT02921971	Completed
			Unknown	IPF	Phase 1/2	NCT01529853	Completed
	Carlumab	CCL2	1 mg/kg, 5 mg/kg, 15 mg/kg	IPF	Phase 2	NCT00786201	Completed
	Olokizumab	IL-6	64 mg	RA	Phase 3	NCT02760407	Completed
			64 mg	RA	Phase 3	NCT02760433	Completed
			64 mg	RA	Phase 3	NCT03120949	Completed
	Clazakizumab	IL-6	Unknown	RA	Phase 2	NCT02015520	Completed
	Vobarilizumab	IL-6 receptor	75 mg, 150 mg, 225 mg	RA	Phase 2	NCT02518620	Completed
	Mirikizumab	IL-23p19	125 mg, 250 mg	PS	Phase 3	NCT03482011	Completed
			125 mg, 250 mg	PS	Phase 3	NCT03535194	Completed
			Unknown	PS	Phase 3	NCT03556202	Completed
	Bimekizumab	IL-17	Unknown	PS	Phase 3	NCT03598790	Active, not recruiting
			Unknown	PS	Phase 3	NCT03766685	Completed
	Secukinumab	IL-17A	300 mg	ALN	Phase 3	NCT04181762	Recruiting
	Tildrakizumab	IL-23p19	Unknown	PsA	Phase 2/3	NCT03552276	Active, not recruiting
			Unknown	PsA	Phase 3	NCT04314544	Recruiting
			Unknown	PsA	Phase 3	NCT04314531	Recruiting
			Unknown	PsA	Phase 3	NCT04991116	Active, not recruiting
	Abatacept	CTLA4	125 mg	PSS	Phase 3	NCT02067910	Completed
			125 mg	PSS	Phase 3	NCT02915159	Completed
	Iscalimab	CD40	Unknown	PSS	Phase 2	NCT03905525	Active, not recruiting
	Ublituximab	CD20	150 mg, 450 mg	RMS	Phase 3	NCT03277261	Completed
			150 mg, 450 mg	RMS	Phase 3	NCT03277248	Completed
			150 mg, 450 mg	RMS	Phase 3	NCT04130997	Enrolling by invitation
	Ianalumab	BAFF receptor	Unknown	SLE	Phase 3	NCT05126277	Recruiting
Chemotherapeutic drugs + senolytic agents	E/C + N	E: topoisomerase-II C: DNA N: bcl-2	E: 100 mg/m^2^ C: 75 mg/m^2^ N: 150 mg	Solid tumors	Phase 1	NCT00878449	Completed
	E + N I + N	E: topoisomerase-II I: topoisomerase I N: bcl-2	E: 150 mg I: 75/180 mg/m^2^ N: unknown	Solid tumors	Phase 1	NCT01009073	Completed
	P + N	P: tubulin N: bcl-2	P: 175 mg/m^2^ N: 150 mg	Solid tumors	Phase 1	NCT00891605	Completed
	D + N	D: microtubule N: bcl-2	D: 75 mg/m^2^ N: 150 mg	Solid tumors	Phase 1	NCT00888108	Completed
	G + N	G: DNA N: bcl-2	G: 1000 mg/m^2^ N: 150/325 mg	Solid tumors	Phase 1	NCT00887757	Completed

*TERT* telomerase, A*AV* adeno-associated virus, *D* dasatinib, *Q* quercetin, *OA* osteoarthritis, *AD* Alzheimer’s disease, *ALS* amyotrophic lateral sclerosis, *AD-MSCs* adipose-derived mesenchymal stem cells, *UC-MSCs* umbilical cord-derived mesenchymal stem cells, *CNS10-NPC-GDNF* human neural progenitor cells expressing glial cell-derived neurotrophic factor, *CKD* chronic kidney disease, *IPF* idiopathic pulmonary fibrosis, *CCR2/CCR5* chemokyne receptor-2 and 5, *CCL2* CC-chemokine ligand 2, *BAFF* B-cell activating factor, *OS* osteoporosis, *PFSSS* pulmonary fibrosis secondary to systemic sclerosis, *SS* systemic sclerosis, *NSLF* nonalcoholic steatohepatitis with liver fibrosis, *SS* systemic sclerosis, *PS* psoriasis, *PsA* psoriatic arthritis, *PSS* primary Sjögren’s syndrome, *RMS* relapsing forms of multiple sclerosis, *ALN* active lupus nephritis, *N* navitoclax, *E* etoposide, *I* irinotecan, *C* cisplatin, *P* paclitaxel, *D* docetaxel, *G* gemcitabine

### Metabolic disorders

#### Diabetes

Diabetes, as a metabolic disorder featured by increased blood glucose, result from defective insulin function, impaired insulin secretion, or both, which has increased mortality in recent years. The known etiological basis of diabetes can provide hints for rejuvenation strategy for treatment. SCs contribute to the pathophysiologies of diabetes via their direct impact on pancreatic β-cell function, involvement in adipose tissue malfunction, and SASP-associated tissue inflammation.^384^ Senolytics for removing SCs and senomorphics capable of attenuating the SASP of SCs can ameliorate diabetes and its complications by alleviating insulin resistance and reducing tissue inflammation.^385^ Awaking immune cells to kill SCs is conducive to improving blood glucose regulation function in diabetes treatment.^386^ The increasing exhaustion and senescence of β cells with age is also the driver of diabetes, and the reversal of senescent β cells and enhancing its function contribute to the treatment of diabetes, such as novel drugs for antagonizing β cell senescence,^387^ regulating metabolism burden to protect β cell function,^388^ the inhibition of islet inflammation,^389^ in vivo reprogramming for β cell regeneration^390^ and epigenetic modifications to rejuvenate senescent β cell, etc.^391^ Despite various rejuvenation approaches for diabetes treatment, most are still primarily in a developing, preclinical stage. Currently, systemic interventions for diabetes treatment seem to be more likely from bench to bedside, which have been explored in the diabetes population. Intensive lifestyle therapy at the workplace has been conducted on the diabetes population, and it exhibited great beneficial effects on improving the diabetes symptoms, such as significant weight loss and conducive changes in fat mass, glycemic controlling, and multiple organ insulin sensitivity, in which the underlying biological mechanism contains muscle NAD^+^ production, SIRTs pathways, mitochondrial activity and adipose tissue remodeling.^392^

#### Obesity

Obesity is characterized as an abnormal or excessive accumulation of fat that presents a health risk. The signal transduction pathways mediating obesity pathophysiology have been widely explored. Currently, a variety of anti-obesity drugs targeting obesity-related regulation pathways have been explored and even under clinical trials, such as melanocortin-4 receptor (MC4R) agonists, neuropeptide y receptor y5 (NPY5R) antagonist and glucagon-like peptide 1 receptor (GLP-1R) agonist, etc. The mechanisms of these interventions against obesity are mainly related to the control of calory intake, glucose balance and thermogenesis.^393^ Inflammation and metabolic disturbances associated with obesity could be caused by cellular senescence, and thus elimination of SCs with senolytic interventions may help improve obesity clinically. The previous reported that, in obese mice, the combination of D + Q reduced circulating inflammatory mediators, increased insulin sensitivity, boosted glucose tolerance, and promoted adipogenesis.^394^ Metabolic disorders in obese are also attributable to adipocyte senescence and unhealthy adipose tissue remodeling, and likewise, senescent adipocyte ablation alleviates adipose tissue inflammation and improves insulin resistance.^395^ All the results suggest that the emerging strategy with the removal of SCs have the potential to address obesity-associated metabolic dysfunction as well as its complication.

### Tissue regeneration

Tissue regeneration refers to the partial regeneration of an organism’s tissue that has been injured by external stimuli, which resulted from the regulation of tissue-residing cells and tissue microenvironment. Cellular rejuvenation for tissue regeneration is devoted to the enhancement of tissue-specific cell function and the regulation microenvironment. The repair of endogenous tissue-specific stem cells was observed in various damaged tissue, like lens stem (LES) cells for lens regeneration after cataract surgery,^396^ activations of Lgr6^+^ EpSCs to generate all cell lineage of skin,^397^ endogenous liver progenitor cell-driven liver regeneration,^398^ etc. These encourage the development of exogenous activation of tissue-residing stem cells to recapitulate damaged tissue, such as biomaterials-based delivery system featured by controlled and sustainable drugs or other biologically active substances release, in osteochondral regeneration, wound healing, and spinal cord repair, and so on.^399^ The dysfunction and senescence of differentiated tissue-specific cells and microenvironment imbalance are other key players that impact tissue regeneration. For example, SCs-matrix interaction is responsible for the difficulty in healing in chronic wounds, because the SASP and ROS production from SCs results in increased matrix proteolysis and inflammation, dysfunction in stem cell, impaired vascular endothelial cells and exacerbated inflammatory microenvironment, and in turn, the microenvironment disturbance further accelerates cellular senescence.^400^ Therefore, the strategies with the elimination of SCs, improvement of tissue cell function, or restoration of microenvironment homeostasis are promising in tissue regeneration. These approaches contain clearance of SCs with senolytics, exogenous stem cell transplantation for replenishing the stem cell pool, EVs and their engineered derivatives for alleviating cellular senescence, and wound inflammatory and chemical compounds for rejuvenating SCs, etc. However, targeting tissue cells and microenvironment fails to achieve the functional regeneration of the large tissue defect or other severe and irreversible tissue damage, such as deep second-degree skin burns, diabetic ulcers, and massive liver defects. Recent advancements in somatic cell reprogramming technology have brought to light the possibility of functional repair of damaged tissue. Impressively, the regeneration of sweat gland cells by epidermal cell, fibroblasts, and MSCs reprogramming not only promotes wound healing in deep second-degree burned skin, but also confers the sweat function, which achieves the functional repair of damaged skin.^401–403^ Ex vivo reprogramming has the disadvantages including the dependence on startling cell sources and potential contamination of in vitro manipulation. Currently, to overcome this obstacle, in situ regeneration based on in vivo reprogramming has developed rapidly, which is applicated to the regeneration of pancreatic β cells, induced cardiomyocyte-like cells, expandable neural stem cells, and sensory hair cells.^184^ Despite the great leap that has been made, in vivo reprogramming is still in its infancy and lots of bottlenecks need to be overcome.^184^ Comprehensively, functional tissue regeneration still remains great challenge facing us.

### Degenerative disorders

#### Bone-degenerative disease

The term “bone-degenerative disease” refers to conditions that progressively deteriorate bones and are primarily defined by degeneration, in which osteoporosis is more prevalent. Clearance of SCs has become lucrative methodology for osteoporosis treatment. Age-related osteoporosis has been demonstrated to be improved by genetic elimination of p16,^404^ and a combination of D and Q also performed well in repairing bone microstructure as seen in radiation-related osteoporosis.^405^ D + Q for the treatment of osteoporosis is being tested in the clinical trial (NCT04313634). Senomorphics that prevent the production of pro-inflammatory proteins, like the JAK inhibitor ruxolitinib, significantly reduce age-related osteoporosis by possibly suppressing certain factors like IL6, IL8, and PAI1.^404^

OA is a degenerative disease of the cartilage that develops with ageing and shares a pathogenesis with the SASP and senescence of joint tissue cells. SASP has been linked to cartilage deterioration, and age-related mitochondrial dysfunction and accompanying oxidative stress may cause senescence in joint tissue cells.^406^ Senolytic agents and senomorphic therapy have potential therapeutic value and implications in OA treatment. Navitoclax and UBX0101 are potential senolytic agents that work to alleviate OA, and the trial of UBX0101 in OA has been completed clinically (NCT03513016). Fisetin is a more selective senolytic candidate with lower hematological toxicity, which can activate SIRT1 to block the inflammatory response in OA chondrocytes,^407^ and Fisetin is now undergoing a clinical trial as a treatment for OA (NCT04210986). Senomorphic agents targeting MMPs can reduce OA symptom, increase type II collagen and inhibit chondrocyte degeneration in WT mice.^408^ IL-6 receptor inhibitor tocilizumab is presently being tested in phase III trial for hand OA (NCT02477059).

#### Neurodegenerative diseases

Alzheimer’s disease (AD), Parkinson’s disease (PD), and amyotrophic lateral sclerosis (ALS) are three of the neurodegenerative diseases. The accumulating evidences have indicated the contributions of cellular senescence to AD pathophysiology, by which telomerase deficiency and telomere shortening, accumulation of β-amyloid (Aβ), tauopathy, and oxidative stress get involved.^409^ Removal of SCs pharmacologically can improve memory of AD by reduction of brain Aβ load and tauopathy. The first-generation senolytic ABT263 is able to clear senescent astrocytes and microglia, modulate tau aggregation and inhibit tau-associated cognitive decline.^410^ Likewise, D + Q senolytic treatment reduced Aβ-related oligodendrocyte senescence, improved cognitive impairments, and decreased Aβ load and neuroinflammation in AD mice.^411^ In another way, D + Q also decreases cortical Tau‐containing neurofibrillary tangles (NFTs) burden, brain atrophy and neuron damage.^412^ However, senolytic therapy is rarely reported in treating PD and ALS. Stem cell-based therapies against neurodegenerative diseases hold promise. MSCs can enhance spatial learning and prevent memory impairment in AD via various mechanisms including reducing Aβ plaques and tau hyperphosphorylation, reversing microglial inflammation, and promoting anti-inflammatory response.^413^ Currently, many clinical trials of MSCs in AD treatment have been approved (e.g., NCT03117738, NCT04040348, and NCT02672306). The transplantation of NSCs with paracrine effect can decrease tau and Aβ levels, alleviate neuroinflammation, improve neurogenesis and enhance cognitive function via releasing neuroprotective or immunomodulatory factors.^414^ Current stem cell sources that have been implicated in PD include NSCs, human ESCs, iPSCs, as well as directly induced dopamine neurons.^415^ In addition, some clinical trials for treating ALS with stem cells were completed and it also gained desired results in various countries, including autologous MSC, endogenous MSC mobilization, NSC (NCT05306457), T-cell vaccination combined with autologous MSC and NSC therapy offers proof-of-concept that stem cell grafting as a therapy for ALS is doable and supports a continuous emphasis on perfecting stem cell-based therapeutic techniques to gain maximum benefit in ALS.^416^ Despite certain results of stem cell therapies that have been achieved neurodegenerative diseases, the disadvantages of this approach including the demand for immunosuppression and a neurosurgery operation, the poor maintenance of stem cell properties, and imperfect cell delivery systems, still need to be solved.

### Cardiovascular diseases

The incidence and prevalence of a wide range of cardiovascular diseases (CVD) increase as a function of age. Molecular pathways involved in cellular senescence, oxidative stress, insulin signaling, autophagy, and inflammation may link the cardiovascular homeostasis deterioration.^417^ Cellular reprogramming is an emerging technology for cardiac regenerative medicine, and it works to treat CVD following three ways: (1) in vitro reprogramming of startling cells such as fibroblasts into cardiomyocytes via initiating cardiac development program, which provides cell source for cardiac regeneration;^418^ (2) in vivo cardiac reprogramming by lineage transcription factors or microRNA manipulation, in which resident cardiac fibroblasts are converted into cardiomyocytes in situ;^419^ (3) Partial reprogramming of cardiomyocytes into a fetal state to restore the function of senescent cells via temporarily reactivating the nascent developmental program in mature cardiomyocytes.^420^ The management of cardiac inflammation, such as targeting inhibition of IL-1 and inflammasome, also work to alleviate CVD, and oral NLRP3 inflammasome inhibitors and IL-1 signaling antagonist including Rilonacept, Canakinumab, and Anakinra have been under the clinical trials.^421^ Senolytics therapy is another potential approach to attenuate or prevent several CVD, which can improve myocardial function and alleviate atherosclerosis by clearance of senescent endothelial cells, senescent foam cells, and senescent T cells.^422^

### Chronic lung diseases

Chronic lung disease (CLD) refers to a spectrum of persistent lung conditions that impair quality of life and are typically resistant to treatment. The conventional therapy regime for CLD treatment with nasal medications, immune-suppressive drugs, and surgery (in severe cases) only supports the decrease of disease progression rate. Cellular rejuvenation strategies based on stem cell therapy have ushered an alternative in the treatment of CLD. People with CLD may benefit from stem cells in the following ways: (1) lowering airway inflammation and preventing additional harm; (2) creating new, healthy lung tissue to replace any damaged tissue in the lungs; (3) promoting the development of new capillaries with tiny blood channels to enhance lung function.^423^ The candidate cell sources rejuvenating CLD include HSCs, ESCs, MSCs, and bronchoalveolar stem cells), and desired efficacy in stem cell therapies promotes more related clinical trials to be developed. Based on the differentiation potential of stem cell therapy, ex vivo lung bioengineering also offers exciting new therapeutic approaches for CLD, which contains physical stimuli such as air ventilation and blood perfusion and stem cell to create a functional lung organ.^424^ In the 3D microenvironment, physical stimuli relevant to lung biology and soluble factors can facilitate the establishment of bioengineered lungs through modulating stem cell fate, which is expected to be a future alternative for transplantation to treat CLD. However, the most important issues to be resolved are how to provide the lung with the ideal conditions of ventilation, perfusion, and oxygenation during the biofabrication process, as well as the best cell types, media, and growth factors (being likely various for the airway and vascular compartments).

### Eye-related diseases

Macular degeneration, often known as age-related macular degeneration (AMD), is a serious eye condition underlined by photoreceptor loss and choriocapillaris and retinal pigment epithelium (RPE) degenerating (CC). The accumulating evidence shows that AMD is mainly attributable to oxidative stress, cellular senescence, and inflammation.^425^ The enhancement of autophagy can resist oxidative damage and mitigate the progression of AMD, and there are potential rejuvenative strategies activating autophagy for the alleviation of AMD, including mTOR inhibitors, hormones, melatonin, and antioxidants in the diet, where the interaction between the NFE2L2, PGC-1, p62, AMPK, and PI3K/Akt/mTOR pathways could be extremely important.^425^ As a result of the role of cellular senescence in the pathogenesis of AMD, it may be possible to use senolytics that kill senescent cells and stop the bystander effects of SASP to treat AMD. Recently, in a phase I study, Unity Biotechnology Company reports that a single dose of UBX1325 improved wet AMD patients’ visual acuity over the course of 24 weeks.

Glaucoma and age-related cataract (ARC) are also chronic, progressive eye diseases, and regeneration and re-functionalization of damaged trabecular meshwork (TM) or lens with stem cells have been regarded as a therapeutic alternative for these two conditions. A number of stem cells, including trabecular meshwork stem cells (TMSCs), ESCs, iPSCs, and MSCs, have demonstrated efficacy in maintaining intraocular pressure (IOP) equilibrium and restoring TM cellularity.^426^ These stem cells can home and integrate into the TM tissue, naturally differentiating into functional TM cells as well as secreting factors that facilitate tissue regeneration.^427^ For ARC treatment, the preservation of endogenous lens epithelial stem/progenitor cells after the extraction of cataractous lens is a new paradigm, which can achieve functional lens regeneration and avoid notable risks of complications caused by the implantation of an artificial intraocular lens.^396^

### Chronic kidney diseases

Chronic kidney disease (CKD) is charactered by persistent kidney function decline, and its progressive nature often results in end-stage renal disease (ESRD). The growing evidences revealed that cellular senescence, stress-induced premature ageing, SASP, oxidative stress, and inflammation-mediated CKD development.^428^ By the selective elimination of SCs, ABT-263 can improve kidney function and stimulate tubular proliferation to reestablish a regenerative phenotype.^429^ Subsequently, a clinical trial suggested that senolytics with D + Q improved physical function in CKD patients (NCT02848131). Various drugs targeting SASP, like glucocorticoids, resveratrol and other protease inhibitors have been reported to attenuate CKD, and most of these mainly inhibit NF-κB signals and reduce ROS.^430^ Stem cell therapy has become the most likely effective therapeutic method to slow and even reverse CKD progression, in which MSC transplantation is widely explored due to their easy collection, low immunogenicity, and high paracrine potential. Both bone marrow MSC and adipose-derived MSC have been shown to have significant renoprotective effects in CKD, including a decrease in fibrosis and glomerulosclerosis as well as an intrarenal inflammatory infiltrate.^431^ The efficacy and safety of using MSC in CKD treatment have been tested in phase I clinical trials, in which two of them using autologous bone marrow MSCs (NCT02166489 and NCT02195323), and others are adipose-derived MSCs (NCT02266394 and NCT01840540). Trials employing MSCs to treat CKD may encounter a number of difficulties, including choosing the best administration method, assuring flourishing, tracking injected cells, immunological rejection, inadequate homing and engraftment, and low immune acceptance.^432^

### Fibrosis

The term fibrosis describes the deposition of fibrous connective tissue as a reparative response to injury or damage, particularly during chronic inflammatory disorders, and Fibrosis leads to tissue architecture disruption, organ malfunction, and eventually organ failure. Based on that known molecular mechanism, numerous cellular rejuvenation strategies to treat fibrosis have been developed. A key contributor to fibrosis is metabolic reprogramming, which includes increased glycolysis, upregulated glutaminolysis, and accelerated fatty acid oxidation.^433–435^ Targeted inhibition of these metabolic processes can regulate collagen production, reduce ECM accumulation and alleviate progression to fibrosis. Miscommunications of macrophage-fibroblast interactions also result in pathological healing and fibrosis, and the restoration of macrophage and fibroblast crosstalk through IGF1 and platelet-derived growth factor C (PDGFC) can reduce tissue dysfunction.^436^ Integrin-mediated TGF-β1 activation is a significant pro-fibrogenic signaling pathway, making it a potentially alluring anti-fibrotic target. Pharmacologically blocking TGF-β1 signaling (e.g., SB431542) or inhibiting integrin signaling (e.g., GSK3008348, PLN-74809, and IDL-2965) have become widely accepted antifibrotic therapy.^437^ Various anti-integrin small molecule agents for idiopathic pulmonary fibrosis (IPF) treatment are under the phase 2 clinical trials (NCT03069989, NCT04072315, and NCT03949530). Strong preclinical studies have identified pro-inflammatory cytokines (e.g., IL-13, CCL2, and CCR2/CCR5) as fibrosis triggers, and likewise, the phase 2 trials of IL-13 inhibitors alone (NCT01266135, NCT00987545, NCT00581997, NCT01872689, and NCT01629667) or IL-4/IL-13 antibodies (NCT02921971 and NCT01529853) have been conducted in the treatment of IPF, skin keloids, and systemic sclerosis. In addition, other clinical trials to treat fibrosis through inhibiting inflammation are also explored, including anti-CCR2/CCR5 (NCT02217475, NCT03028740, NCT03059446, and NCT02330549) for liver fibrosis and non-alcoholic steatohepatitis, anti-IL-1 (NCT01538719) for systemic sclerosis, anti-IL-6 (NCT02453256) for scleroderma, and anti-CCL2 (NCT00786201) for pulmonary fibrosis. During the process of fibrosis, myofibroblasts exhibit remarkable inter-lineage plasticity, which provides opportunities for cellular reprogramming in fibrosis treatment. During cutaneous wound healing, neogenic hair follicles-derived BMP signaling can reprogram myofibroblasts into adipocytes.^438^ Correspondingly, the BMP pathway activation may be the preventive approach to reduce fibrosis and achieve scarless wound healing. Finally, in vivo reprogramming induces myofibroblasts into other functional desired cells, which can not only attenuate fibrosis and also realize in situ tissue repair.^184^

### Inflammation and autoimmune disorders

The strong evidences link chronic inflammation to autoimmune disease pathogenesis, including rheumatoid arthritis (RA), systemic lupus erythematosus (SLE), multiple sclerosis (MS), inflammatory bowel disease (IBD), psoriatic arthritis (PsA), primary Sjögren syndrome (PSS) and psoriasis, etc. Inflammatory cytokines (e.g., TNF-ɑ, IL-17, and IL-23), T cell-mediated inflammatory responses, and B cells-mediated antibody production play deleterious roles in the inflammation regulation on autoimmune diseases, which raises the potential for cellular rejuvenation targeted immunotherapy for autoimmune disease.^439^ Anti-cytokine therapy has revolutionized autoimmune disorders treatment. Targeting TNF-ɑ therapy including monoclonal antibodies (e.g., infliximab) and one receptor-Fc fusion protein (etanercept) is currently availability for RA, psoriasis, and IBD treatment. Inhibition of IL signaling also has been developed against autoimmune diseases, such as IL-1R antagonist (Anakinra), IL-1R1-IgG Fc (Rilonacept), anti-IL-1β mAb (Canakinumab), anti-IL-6 mAb (Sirukumab, Olokizumab, Clazakizumab, and Siltuximab), anti-IL-6R mAb (Tocilizumab, Sarilumab, and Vobarilizumab), anti-IL-17 mAb (Ixekizumab and Secukinumab), anti-IL-17R mAb (Brodalumab), anti-IL17A/F mAb (Bimekizumab), anti-IL-23/p40 mAb (Ustekinumab) and anti-IL-23/p19 mAb (Guselkumab, Risankizumab, Tildrakizumab, and Mirikizumab). Thereinto, monoclonal antibodies targeting IL-6 (Olokizumab: NCT02760407, NCT02760433, and NCT03120949; Clazakizumab: NCT02015520; Vobarilizumab: NCT02518620) have been registered for RA treatment, and other includes anti-IL-23 mAb (Mirikizumab: NCT03482011, NCT03535194, NCT03556202) and anti-IL17A/F mAb (Bimekizumab: NCT03598790 and NCT03766685) for psoriasis treatment, anti-IL-17 mAb (Secukinumab: NCT04181762) for SLE treatment, and anti-IL-23 mAb (Tildrakizumab: NCT03552276, NCT04314544, NCT04314531, NCT04991116) for PsA treatment. Immunosuppression of T cells activation by CTLA4-IgG1 Fc (Abatacept) and anti-CD40 mAb (Iscalimab) in autoimmune diseases also has been studied. Now, Abatacept (NCT02067910 and NCT02915159) and Iscalimab (NCT03905525) have been tested in phase 2 trials for PSS treatment. B cell depletion therapies show great potential for the treatment of autoimmune diseases, and likewise, a large number of monoclonal antibodies targeting B cells function also has been developed, such as anti-CD20 mAb (Rituximab, Ocrelizumab, Ofatumumab, and Ublituximab), anti-CD19 mAb (Inebilizumab), anti-BAFF mAb (Belimumab) and anti-BAFF-R mAb (Ianalumab), in which Ublituximab for MS (NCT03277261, NCT03277248, NCT04130997) and Ianalumab for SLE (NCT05126277) are under the phase 2 trials. Treatment and clinical results for people with autoimmune diseases have been changed by improvements in tailored immunotherapy, but immunosuppression based on decreasing inflammation may lead to serious side effects, such as increasing the risk of infection.^440^ Therefore, future researches seek to sustain immune defenses while inducing lifelong immunological tolerance.

### Other ageing and age-related diseases

Other ageing and age-related diseases are also matters of great concern, including skin ageing, skeletal muscle ageing, reproductive system ageing, and ageing-related hearing loss. Skin ageing is currently a very concerning field, and the reversal or delay of skin ageing has a great market prospect. The process of skin ageing is multifactorial and is influenced by both intrinsic (such as time, genetics, and hormones) and extrinsic (such as UV exposure, and pollution) variables. Senescent skin cells, SASP, oxidative stress, inflammation, and autophagy, all mediate skin ageing pathophysiology. Fu et al. reported the phenomenon that epidermal cells can de-differentiation into stem cells in vivo during wound healing, and this discovery was the first to reveal that adult cells can become adult stem cells, which suggested that the high plasticity of epidermal cells and cellular fate was not one-street.^441^ The subsequent work of Fu et al. demonstrated that dedifferentiation-derived cells cultivated in vitro shared phenotypic and functional traits with EpSCs.^442^ Much more importantly, Fu et al. further discovered that aged epidermal cells had the ability to dedifferentiate into EpSCs, a process for which activation of the Wnt/-catenin pathway was crucial.^443^ Such breakthroughs not only make it possible to reverse skin ageing, but also promote the development of wound repair greatly. Currently, topical cosmetics like sunscreen, retinoids, or resveratrol are the foundation of current strategies for combating skin ageing. However, it is unclear how these products’ effectiveness in preventing skin ageing is related. Anti-ageing drugs such as senolytics or senomorphics may be useful for skin rejuvenation, because such drugs have anti-inflammatory and melanogenic properties that help protect the skin, prevent carcinogenesis, delay ageing, and reduce age-related diseases.^444^ Application of antioxidants to reduce and neutralize free radicals is an alternative method to slow skin ageing. The administration of natural products, especially polyphenols with known antioxidative properties, show the ability to enhance or remove the undesirable signs of ageing skin. Evidence-based knowledge suggests that the topical or oral use of various polyphenol-rich plants can prevent or reduce, besides others, undesirable conditions of skin ageing.^445^ Enhancing skin cell function and remodeling elastin networks hold promise for the restoration of skin youthful state. The developed strategies include stem cells and related EVs,^446^ pharmacological activation of autophagy,^447^ maintenance of circadian rhythmicity on skin-resided stem cell^448^, and regulation of metabolism homeostasis to restore mitochondrial integrity and metabolic output.^449^ Identifying key molecules involved in skin ageing makes great significance to skin rejuvenation. The role of COL17A1 in EpSC competition to orchestrate skin homeostasis and ageing and COL17A1 overexpression against skin ageing also were revealed.^450^ Therefore, targeting activation of COL17A1 is the potential angle for skin anti-ageing therapeutic intervention. Y27632 and apocynin have been identified as COL17A1-inducing drugs, which show the capacity to facilitate skin regeneration and reduce skin ageing.^450^ Similarly, the miR-31-CLOCK-ERK pathway is a significant contributor to and therapeutic target for skin ageing. By inhibiting this pathway, either through conditional miR-31 ablation or with clinically proven MAPK/ERK inhibitors, one can safely and effectively delay the ageing process of the skin.^451^ The molecular mechanisms of skin ageing provide the biological basis for the application of drug-loaded biomaterials to skin rejuvenation. For example, using tetrahedral framework DNA (tFNA) to design a novel bio-switchable miRNA inhibitor delivery system (BiRDS), which can fuse miR inhibitors within the framework and maximize the loading ability. MiR-31 inhibitors-loaded BiRDS have excellent skin penetration and high RNA delivery efficiency, significantly combating skin ageing.^452^

## Utilizing rejuvenation for cancer treatment

### Cancer and its relation to rejuvenation

While most studies have focused on the function of senescence in non-cancer cells, it is clear that cancer cells may also establish a senescence response, which makes it possible to exploit senescence to treat cancer. Induction of senescence in tumors includes chemotherapy, radiotherapy, cell cycle inhibition, and telomerase inhibition. However, persistently therapy-induced senescence (TIS) may create a pro-inflammatory microenvironment.^453^ Meanwhile, SASP can contribute to angiogenesis to facilitate tumor development and an EMT in neighboring cancer cells and induce ECM degradation further resulting in the migration and metastasis of cancer cells.^454^ To overcome this obstacle, a sequential therapy regimen consisting of TIS followed by a second compound that specifically kills senescent cancer cells (senolytic agent) is highlighted as a one-two-punch method for treating cancer. For example, navitoclax has shown desired results when combined with various TIS, including ionizing radiation, rituximab (CD20 antibody), doxorubicin, paclitaxel, docetaxel, or gemcitabine.^455^ A phase II study (NCT01087151) demonstrated that, in patients with chronic lymphocytic leukemia, rituximab plus navitoclax exhibit higher effectiveness, prolonged progression-free survival, and well tolerance than that of rituximab monotherapy.^456^ Other chemotherapeutic agents like etoposide (NCT00878449), irinotecan (NCT01009073), cisplatin (NCT00878449), paclitaxel (NCT00891605), docetaxel (NCT00888108), and gemcitabine (NCT00887757) that are combined with navitoclax also have been tested to treat various cancers. For sequential treatment regimen in cancer, the issue that need to be solved is identifying pro-senescence drugs with high efficacy and selectivity in inducing cancer cell senescence.

### Cellular reprogramming strategy converting malignancy to benignity

**Fig. 6:**
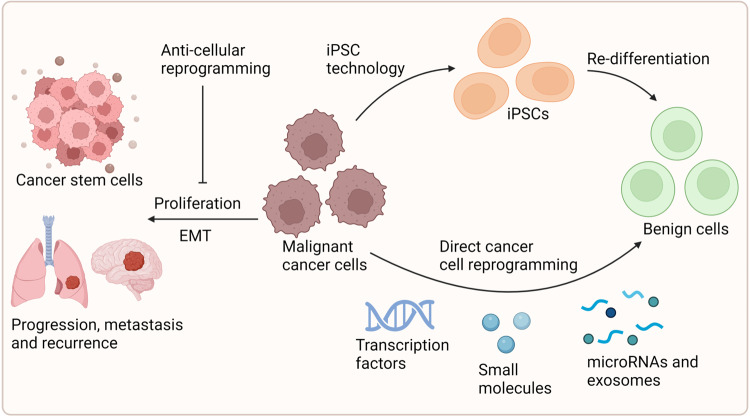
The mechanisms for cellular reprogramming in cancer. Cancer cell reprogramming treatment aims to convert the malignancy to benignity or provide a therapeutic target to inhibit the formation of CSCs. Yamanaka factors-mediated iPSCs technology has been recognized as a common method for the conversion of cancer cells to benign pluripotent cells, and cancer cells-derived iPSCs can re-differentiate into functional cells with less malignancy and free of tumorigenic potential. Cancer cells also can be directly reprogrammed into benign cells via various reprogramming strategies, such as lineage-specific factors, small molecules, microRNAs, and exosomes. Responsive cellular reprogramming based on EMT contributes to CSCs formation, which mediates the initial, progression, metastasis, and post-treatment recurrence. The role of cellular reprogramming in the formation of CSCs suggests that anti-cellular reprogramming strategies may be considered as a therapeutic alternative in cancer treatment. EMT Epithelial–mesenchymal transition. Created with BioRender.com

It is well recognized that cancer cells can develop a malignant transition from benign cells, but the question that whether it is possible to genetically and epigenetically transform malignant cancer cells to benignity is interesting. The discovery of somatic cell reprogramming encourages the idea that cancer cells, featured by genetic and epigenetic plasticity, can rescue benign cell functions by cellular reprogramming theoretically. Cell reprogramming techniques that trigger the switch from malignancy to benignity include transcription factors, chemical cocktails, microRNAs, and exosomes (Fig. 6).^457^ Transcription factor-mediated cancer cell reprogramming is a pioneering strategy to retrieve benign functions. The conversion of cancer cells to iPSCs with benign phenotypes has been frequently studied. With the induction of pluripotency-associated transcription factors, melanoma cells, hepatoma cells, and colorectal cancer cells, lung adenocarcinoma and gastrointestinal cancer cell can be reprogrammed into iPSCs, and these cancer cells-derived iPSCs maintain benignity without the formation of a visible tumor in vivo.^266^ In addition, the cancer cells-derived iPSCs can undergo multi-lineage differentiation, such as epithelial and mesenchymal cells as well as neuron. More importantly, these differentiated cancer cells are less aggressive and lack the ability to form tumors than their parental cancer cells. Lineage-specific factors manipulation also has shown proven capabilities to directly convert cancer cells to desired functional cells without the acquisition of pluripotency. For example, overexpression of the transcription factor CCAAT/enhancer-binding protein alpha (C/EBPα) has been utilized to successfully transform human lymphoma and leukemia B cell lines into macrophage-like cells, with approximately 80% efficiency, and the reprogrammed cells exhibited poor tumorigenicity in vivo.^458^ Likewise, hepatocellular carcinoma can be directly induced into hepatocyte-like cells with normal functions when treated with the combinatorial manipulation of hepatocyte transcription factors.^459^ Although transcription factor-mediated cellular reprogramming is feasible and ethically acceptable in cancer treatment, such technology has additional challenges including cost and delivery efficiency, and in vivo procedure. The urgent need for developing optional methods is driven by safety and effectiveness concerns brought on by genetic manipulations. The chemical reprogramming technology based on small molecules has some particular advantages, such as affordability, ease of use, versatility that is easily programmable, permeability, and reversibility.^460^ Small molecule cocktail (SMC) consisted of SB431542 (TGFβ inhibitor), CHIR99021 (GSK3β inhibitor), BIX01294 (H3K9 methyltransferase/G9a inhibitor), and all-trans retinoic acid (ATRA), could give rise to losing malignant phenotype and acquiring hepatocyte properties in various liver cancer cells.^461^ MicroRNA and exosome delivery are novel anti-cancer alternatives in a manner of cellular reprogramming. Lin et al. reported using microRNA-302s could convert skin cancer cells into iPSCs with decreased tumorigenicity and genomic demethylation. These pluripotent cancer cells displayed more than 86% gene expression similarity to human ESC lines.^462^ Under the stimulation of lineage-specific factors, such cells could differentiate into functional cells with benignity properties, like neurons and chondrocytes. ESCs-derived exosomes also deliver ESC-related reprogramming factors to convert malignant cells to benignity ones.^463^ However, the precise and efficient regulation of cellular reprogramming to malignancy conversion with microRNA or exosomes faces obstacles, and few investigations have been conducted in the extending frontiers.

### Anti-cellular reprogramming therapy for cancer treatment

The increasing evidence suggests that cellular reprogramming also contributes to cancer initiation, development, and recurrence (Fig. 6). Specific transcription factors silence- or epigenetic gene mutation-mediated cellular reprogramming initiate cancer initiation. For example, the depletion of PTEN in NSCs could activate Paired Box 7 (PAX7) and induce the generation of glioblastoma stem cell-like cells, which can lead to intracranial tumors in vivo.^464^ Cellular reprogramming mediates cancer development through cancer cell trans-differentiation or dedifferentiation. In non-small cell lung cancer, Lkb1 deficiency induced the trans-differentiation of adenocarcinoma cells into squamous cell carcinoma.^465^ In prostate cancer, luminal adenocarcinoma cells may trans-differentiate into neuroendocrine cells as a result of TP53 and PTEN depletion.^466^ Cancer cells can be reprogrammed by EMT and cell fusion to acquire stemness and differentiate into cancer stem cells (CSCs), which is a crucial step in cancer development. For example, single-cell analysis of organoids derived from CD44^+^ colorectal CSCs revealed that TWIST1-mediated EMT played an essential role in inducing cancer cell differentiation, and external stimulation with TGF-β1 can initiate EMT and promote the generation of CD44^+^ CSCs.^467^ Cellular reprogramming generating CSCs also contributes to therapy resistance and the recurrence of cancers, which may be the biological basis of poor clinical outcomes and post-treatment cancer recurrence. Conventional anti-cancer therapeutics, including chemotherapy and radiotherapy, enable cancer cells to become CSCs. For example, in non-small cell lung cancer, cisplatin-based chemotherapy renders p53 inactive and induces Tribbles Pseudokinase 1 expression, which leads to stemness activation in cancer cells.^468^ Therefore, cisplatin plus histone deacetylase inhibitor may synergistically suppress non-small cell lung cancer progression. Anti-angiogenesis therapy also has a great deal of potential in treating cancer, and antiangiogenic agents drive CSCs reprogramming by generating tumor hypoxia microenvironment.^469^ CSCs reprogramming also has been observed in immunotherapy, such as adoptive cell transfer (ACT) therapy. In melanomas, T cell-driven inflammatory stimuli lead to melanomas cells with the transition of the differentiated and dedifferentiated state and confer the ACT resistance.^470^ Collectively, cellular reprogramming is the key player in cancer initiation, development, recurrence, and therapeutic resistance. Synergistic therapy of conventional therapeutics and anti-cellular reprogramming may represent a promising strategy in cancer treatment. In the future, identifying molecular mechanisms involved in CSCs reprogramming is of great significance to anti-cellular reprogramming therapy.

## Summary

The discovery of epidermal cell de-differentiation into stem cells and somatic cell reprogramming into iPSCs revealed that cell fate is not a one-way street. Terminally differentiated cells can reset their genetic and epigenetic properties and acquire the undifferentiated phenotype, and likewise, SCs can also get rid of senescence-related signatures and restore the youthful state. Emerged evidence has indicated the important contribution of cellular senescence in ageing and ageing-related diseases, which encourages the hypothesis that the reversal of ageing may be possible, and targeting cellular senescence may pave the way for that. The development of various cellular rejuvenation strategies provides compelling evidences that the ageing process is not irreversible. Particularly, the effectiveness of stem cell therapy and DR has been tested in the real world, yielding to desired results. Hopefully, there is a great possibility of translation of these rejuvenation strategies to address human ageing, age-associated diseases, and cancers. Therefore, it is reasonable to expect that clinical rejuvenation approaches to treat ageing-related diseases and even to reverse ageing will be boomed within the next two or three decades.

## Conclusions and future perspectives

Throughout human history, the quest to extend lifespan or restore youthful state has been relentless. With the enormous progress in medical technology, including a deeper understanding of cell senescence and age-associated pathophysiology, it has become plausible to extend the human lifespan while preserving health. It is extremely essential to gain insight into the basic principles regulating cellular rejuvenation. Although many aged phenotypic and functional characteristics are studied, the formation, maintenance, and functional contribution to the disease process of aged cells still need further exploration. The study of cellular rejuvenation targeting ageing-related mechanisms is promising to develop potential therapeutic interventions for postponing and reversing ageing, and treating ageing-related diseases.

For decades, one of the dominant theories in ageing research has been that ageing results from the accumulation of DNA changes, mainly genetic mutations, which prevent more and more genes from functioning properly over time. These malfunctions, in turn, can cause cells to lose their properties, leading to the breakdown of tissues and organs and ultimately to ageing and diseases. However, the emerging evidences claim that epigenetic information loss over time is the major cause of mammalian ageing, and epigenetic regulation can restore youthful gene expression patterns. Current advances have provided a comprehensive and detailed multi-level epigenetic panorama of ageing, elucidated some common and unique characteristics of physiological ageing and ageing-related diseases, identified novel biomarkers of ageing, and revealed new mechanisms of epigenetic remodeling in cell and organ ageing. It is expected that future research on ageing epigenetic inheritance will further expand the relationship between chromatin three-dimensional structure and function. Especially, with the development of epigenetic editing technology, scientists can make specific perturbations to the epigenome, to distinguish the causal relationship between the three-dimensional structure of chromatin and cell function. Epigenetic changes caused by ageing in more diverse cell types and pathophysiological states should also be extensively explored to discover conditional regulation mechanisms of ageing. For epigenetic rejuvenation, developing safe and stable strategies that modulate the epigenetic landscape of aged cells to a primitive state are important for cells to exert rejuvenating effects without cancer risk. Furthermore, systematic comparisons of epigenetic dynamics during ageing and partial reprogramming will contribute to identifying key checkpoints for reversing the ageing process and inform the design of potential intervention strategies for ageing-related diseases.

Pathological accumulation of SCs is also associated with ageing and a range of diseases, and SCs may be potential pharmacological targets for delaying the ageing process. In respect of targeting SCs, there are still many potential markers, like chromatin dynamics and transcriptional signaling, and pharmacological interventions deserving exploitation, to effectively regulate the secretory phenotype of SCs. SC elimination and SASP inhibition have shown some efficacy in clinical studies of treating functional degeneration and chronic diseases in ageing. Notably, SC populations are heterogeneous in terms of composition, function, and tissue distribution, even among species, which is also a problem in the transition from laboratory to clinic. Therefore, identifying the cell or organ-specific markers of SCs is of great guiding significance for future clinical treatment. Targeting the cell microenvironment and systemic signals makes sense for tissue-specific cell and organism rejuvenation. Stem cells play a crucial role in maintaining tissue homeostasis, and cell microenvironments also regulate stem cell behavior, which together form a regenerative unit. External signals from the ageing microenvironment appear to dominate the intrinsic function of young stem cells. In contrast, signals from the young microenvironment may have a limited effect on the regeneration of aged stem cells. It would be interesting to identify the genes or pathways that make aged stem cells insensitive to external signals in young microenvironment. Although many clinical trials registered for stem cell treatments, an effective and safe stem cell therapy to slow or reverse tissue ageing has not yet been identified. Several obstacles still need to be overcome, including proper differentiation and integration of cells in tissues, maintenance of the youth of stem cells and their progeny in ageing tissues, and prevention of tumorigenesis. It will be important to determine the specific mechanisms, which have the potential to provide better treatment pathways-using stem cell transplantation or utilizing endogenous stem cell banks. Recent advances in single-cell transcriptomics and pedigree tracing techniques provide a systematic understanding of stem cell ageing mechanisms. Developing and utilizing new techniques to track and manipulate stem cells will still be a key field. The systematic identification of gene networks, involved in functional changes, age-dependent changes in RNA and protein and metabolite molecules, and cellular interactions, will contribute to further studies on stem cells in tissue repair and ageing-related diseases. In addition, rejuvenating strong circadian rhythms can optimize organismal physiology and avoid the risk of disease to prolong health. Clinically, circadian rhythms including a series of cyclical life activities, such as diet, sleep and exercise, vary from person to person. It is of great clinical value to develop methods to predict individual circadian rhythms. Machine learning and artificial intelligence methods may help to identify a series of biomarkers to predict circadian rhythms, which has great application prospects for determining the optimal biological clock pattern for each individual.

Despite the great progress in cellular rejuvenation, the potential limitations have led to cellular rejuvenation rarely being tested in human studies. Cellular rejuvenation for reversing ageing and age-related diseases as well as cancers has been extensively studied. While cellular rejuvenation holds great promise, key questions remain to be addressed. (1) Cellular reprogramming strategy can reverse age-related physiological changes and promote tissue regeneration by resetting the epigenetic clock and changing cell fate, but the problems such as relatively low reprogramming efficiency and potential safety concerns, remain the obstacle in the path of its application. (2) The pharmacological delivery system is difficult to express the pluripotency factors with high efficacy. The toxicity might be induced by drug combinations, then reducing the effectiveness of the cocktail and causing side effects in normal cells. (3) Clearance of SCs and decreasing SASP exert a beneficial effect on organ repair and disease treatment, but poor cell selectivity of senolytics may result in the damage of normal tissue, and SASP inhibitors targeting specific secretory factors also have limited therapeutic effects on multiple factors-mediated diseases. Besides, completely senescence-specific markers are still absent. (4) Stem cell therapy as a rejuvenative strategy holds great promise in the reversal of ageing and alleviation of diseases. Despite the advances in many clinical trials of stem cell therapy, optimizing in vitro culture environment, improving the delivery system of stem cells, and reducing immune rejection are still the major challenges to obtain high-quality stem cells and enhance that therapeutic effect. (5) Restoring defective intercellular communications by the inhibition of inflammation can rejuvenate ageing-impaired changes, but long-term inflammation inhibition may lead to immunosuppression.

Collectively, cellular rejuvenation holds great promise for preventing and treating ageing-related diseases from different dimensions. A healthy and rejuvenated state of the organism can maintain stable characteristics and biological functions without excessive ageing-related degeneration or deterioration. Of note, system therapies such as DR and exercise are available rejuvenation strategies mostly closed to bedside, whose mechanism at the cellular level and molecular level has been expounded.^471^ The role of DR in health and diseases has been discussed above. Exercise greatly activates the immune system, facilitates DNA repair processes, maintains metabolism homeostasis, and is capable of lowering the risk of diabetes, obesity, cancer, osteoporosis, AD, and depression as well as prolonging the lifespan.^472^ The exploration of diet and exercise programs for different populations is the direction that needs to be paid attention to in the future.
